# A visual approach for analysis and inference of molecular activity spaces

**DOI:** 10.1186/s13321-019-0386-z

**Published:** 2019-10-22

**Authors:** Samina Kausar, Andre O. Falcao

**Affiliations:** 1 LaSIGE, Departamento de Informática, Faculdade de Ciências, Universidade de Lisboa, 1749-016 Lisboa, Portugal; 2 BioISI: Biosystems & Integrative Sciences Institute, Faculdade de Ciencias, Universidade de Lisboa, 1749-016 Lisboa, Portugal

**Keywords:** Structure activity relationship (SAR), Molecular/chemical space, Two dimensional kernel density estimation, Noncontiguous atom matching structural similarity function (NAMS), t-SNE, PCooA, Non-metric MDS, Sammon mapping

## Abstract

**Background:**

Molecular space visualization can help to explore the diversity of large heterogeneous chemical data, which ultimately may increase the understanding of structure-activity relationships (SAR) in drug discovery projects. Visual SAR analysis can therefore be useful for library design, chemical classification for their biological evaluation and virtual screening for the selection of compounds for synthesis or in vitro testing. As such, computational approaches for molecular space visualization have become an important issue in cheminformatics research. The proposed approach uses molecular similarity as the sole input for computing a probabilistic surface of molecular activity (PSMA). This similarity matrix is transformed in 2D using different dimension reduction algorithms (Principal Coordinates Analysis ( PCooA), Kruskal multidimensional scaling, Sammon mapping and t-SNE). From this projection, a kernel density function is applied to compute the probability of activity for each coordinate in the new projected space.

**Results:**

This methodology was tested over four different quantitative structure-activity relationship (QSAR) binary classification data sets and the PSMAs were computed for each. The generated maps showed internal consistency with active molecules grouped together for all data sets and all dimensionality reduction algorithms. To validate the quality of the generated maps, the 2D coordinates of test molecules were computed into the new reference space using a data transformation matrix. In total sixteen PSMAs were built, and their performance was assessed using the Area Under Curve (AUC) and the Matthews Coefficient Correlation (MCC). For the best projections for each data set, AUC testing results ranged from 0.87 to 0.98 and the MCC scores ranged from 0.33 to 0.77, suggesting this methodology can validly capture the complexities of the molecular activity space. All four mapping functions provided generally good results yet the overall performance of PCooA and t-SNE was slightly better than Sammon mapping and Kruskal multidimensional scaling.

**Conclusions:**

Our result showed that by using an appropriate combination of metric space representation and dimensionality reduction applied over metric spaces it is possible to produce a visual PSMA for which its consistency has been validated by using this map as a classification model. The produced maps can be used as prediction tools as it is simple to project any molecule into this new reference space as long as the similarities to the molecules used to compute the initial similarity matrix can be computed.

## Introduction

Chemical/molecular space reflects high dimensional conceptual spaces that describe the structural diversity of all possible potential pharmacologically active molecules. The size of molecular space is not well defined, yet a fraction of it ranging from thousands to millions of compounds is stored in small molecule databases. Consequently, a part of the huge molecular space is mainly focused to explore the complexity of a relevant small set of chemical structures in many different problems during drug design [1–3]. Nonetheless, molecular space interactive analysis and visualization can serve as a strong tool to explore the diversity of millions of compounds stored in public databases and can increase the performance of drug discovery process. For example, nearest neighbour searches in various defined property regions in molecular space (activity space map) can identify interesting similar molecules (potent analogues) with similar properties [1, 2, 4, 5].

Molecular space visualization methods require that molecules are projected into a reduced set of dimensions (most of the times, two or three) in such a way that the relative distances between molecules are better preserved in this new projected space. As distances should be preserved, molecules with similar activity profiles should appear clustered together [1, 6]. Thus, molecular space visual analysis combines the concept of molecular structure and activity similarity [6, 7]. Since molecular dis/similarity is defined through pairwise distances between projected molecules in reference space, an appropriate choice of a molecular metric space (spatial) representation is crucial for reliable application of molecular spacial analysis. A molecule in metric space is defined as a set of distances computed from the similarity between that molecule to all the other molecules in a given chemical data set. For this purpose, many methods are available in literature to compute dis/similarity. A variety of methods uses either molecular descriptors or fingerprints, which represent different physico-chemical or structural characteristics [8–16]. These approaches entail that each molecule is initially reduced into a vector space by computing a set of attributes, that can be used to infer distance, yet this is not always required as other independent approaches like molecular graph matching approaches can also be used for a direct assessment of structural similarity [17–20].

In metric space representation, a set of *M* molecules is represented in *M* dimensions, as the distance to all the other elements of the set (including itself) must be present. As such, the visualization of this *M*-dimensional metric space in reduced spatial dimensionality is a challenge in data diversity analysis [7, 21, 22]. To address this issue many linear and non-linear approaches have been developed to reduce the dimensionality and complexity of molecular space [1, 6, 21, 23]. In all dimension reduction (DR) methods, the most important characteristic is the optimization of the criterion that guides dimensionality reduction. Since the concept of DR is mainly based on data geometrical representation where data is interpreted as discrete points/objects, the main objective to explore or analyze such geometrical spaces is to discover the relationships between the points within this complex structure of data (manifold) [22]. The main criterion that needs to be optimized in DR algorithms for metric space data is the approximation of the original intermolecular distances (proximity relationships) in the new projection space; DR approaches that are based on optimization of this dimensionality reduction criterion in a linear/non-linear way are collectively referred as distance-preserving approaches [22]. Principal component analysis (PCA) [24], is by far the most common method [1, 25, 26] used in DR, yet it does not fall into this category, as the main purpose of PCA is to represent in less dimensions the linear components that maximize the data variance, not necessarily preserving the distances between data. On the other hand Principal Coordinates Analysis (PCooA) [27, 28], Sammon mapping [29], stochastic neighbor embedding [30] or stochastic proximity embedding [31], to name but a few are distance-preserving DR algorithms and some topology-preserving methods like self-organizing maps [32] or generative topographic mapping (GTM) [33] have been used in cheminformatics [23]. The latter method appears as an interesting approach to reduce dimensions while producing a kernel based probabilistic map, nonetheless it is not as accurate for preserving distances and requires a set of descriptors selected beforehand for data processing [33]. Most of the times, non-linear methods are usually preferred because linear algorithms may be limited to linear projection functions and therefore may not adequately handle complex associations that may be present in such problems [22].

Distance preserving DR methods can then make it possible to project molecules into a 2D reduced molecular space while preserving the original proximity (distances) of molecules as best as possible, assuming that there is always going to be a loss of information as the original molecular space should have a much higher dimensionality. To establish a structure-activity relationship, molecular activity surface maps mostly referred as “activity landscapes” are generated from 2D projected space (reference space) of molecules by adding a property of each molecule as a third dimension [6, 23, 34]. In such projections, the activity of molecules added as third dimension in the projected molecular space is the basis for fitting a generated surface that represents the activity magnitude. Since data is largely scattered in projected space, an interpolation algorithm [35, 36] is required to make a coherent surface onto this 2D projected map. Ideally, structurally similar molecules should appear grouped together in well-separated clusters and each group should have similar properties. This property may not always hold, and that is the case of “activity cliffs”, projected regions that exhibit similar molecules with largely varying activity very close together [9, 16, 35, 37]. Despite these challenges, such analysis may provide a global picture of the spatial characteristics of a given data set.

The descriptive and predictive accuracy of molecular space visualization approaches largely depends upon three main issues, including a) a choice of a molecular space representation, b) the accuracy of DR methods and c) the performance of the interpolation algorithm to generate well estimated activity surface from sparsely projected molecules. To this end, in our approach for visual characterization of molecules in conceptual spaces, a reliable pipeline is generated that can efficiently be used to build a probabilistic surface of molecular activity (PSMA), which can help to understand SAR in different situations. We have thus integrated the advantages of the following different methods in the proposed molecular space mapping approach:

- Choice of molecular space representation: Molecular pairwise similarity was quantified using a graph matching algorithm: The Non-contiguous Atom Matching Structural similarity (NAMS) [17], which is a robust metric space representation method. This algorithm has a higher discriminative power for very similar molecules over other structural or graph matching approaches [17, 38]. However, any other similarity computation method can be used.
- DR methods: We applied four non-linear DR methods including Principal Coordinates Analysis (PCooA) [27], Kruskal Multidimensional Scaling (KMDS) [28], Sammon mapping (SM), [29] and t-Distributed Stochastic Neighbor Embedding (t-SNE) [39].
- PSMA: Non-parametric 2D kernel density estimation (KDE) function [40] created within a Bayesian framework was used to map the most likely activity regions (activity surfaces) from sparsely distributed active and inactive compounds.

This approach is, to our knowledge, new and allows building a non-parametric model out of raw similarity data, which is useful for visualization and has clear predictive properties. This model does not make any assumptions on its form and, due to its construction process, its resulting surface is independent of the coordinate axes. Furthermore, t-SNE applications in molecular space diversity analysis are not a common practice in cheminformatics. A survey of recent literature showed only one work to visualize molecular space using this algorithm [41]. However, under this particular domain, this is a first effort to build activity spatial classification model using this algorithm by comparing its performance with other commonly used tools. Another novel point the present approach tried to address was the use of 2 dimensional KDE for model making. KDE is considered a powerful tool in statistics for truthful assessment of data actual distribution/characteristics [40]. In cheminformatics literature KDE has been used as a robust method to define applicability domains of quantitative structure-activity relationship (QSAR) predictive models [42–44]. Applicability domains are used to define a boundaries in molecular space within which new predictions of QSAR models are considered reliable [45]. We extended the same concept to computing probability density function for active and inactive molecules within 2D projected space and surface was generated from the 2D map containing high promising regions of active molecules. In the presented methodology, integration of KDE in SAR spatial visualization is a new addition in the efforts of molecular space analysis. It must be made clear that, despite the fact that we are using a Bayesian approach to compute the PSMA, our method does not assume independence of the projected coordinates, thus being closer to a full Bayesian classifier than to a naive-Bayes approach. We are thus computing the full 2D probability map and not the individual probability distribution functions of each coordinate axis.

## Methodology

### Overview of the methodology

**Fig. 1:**
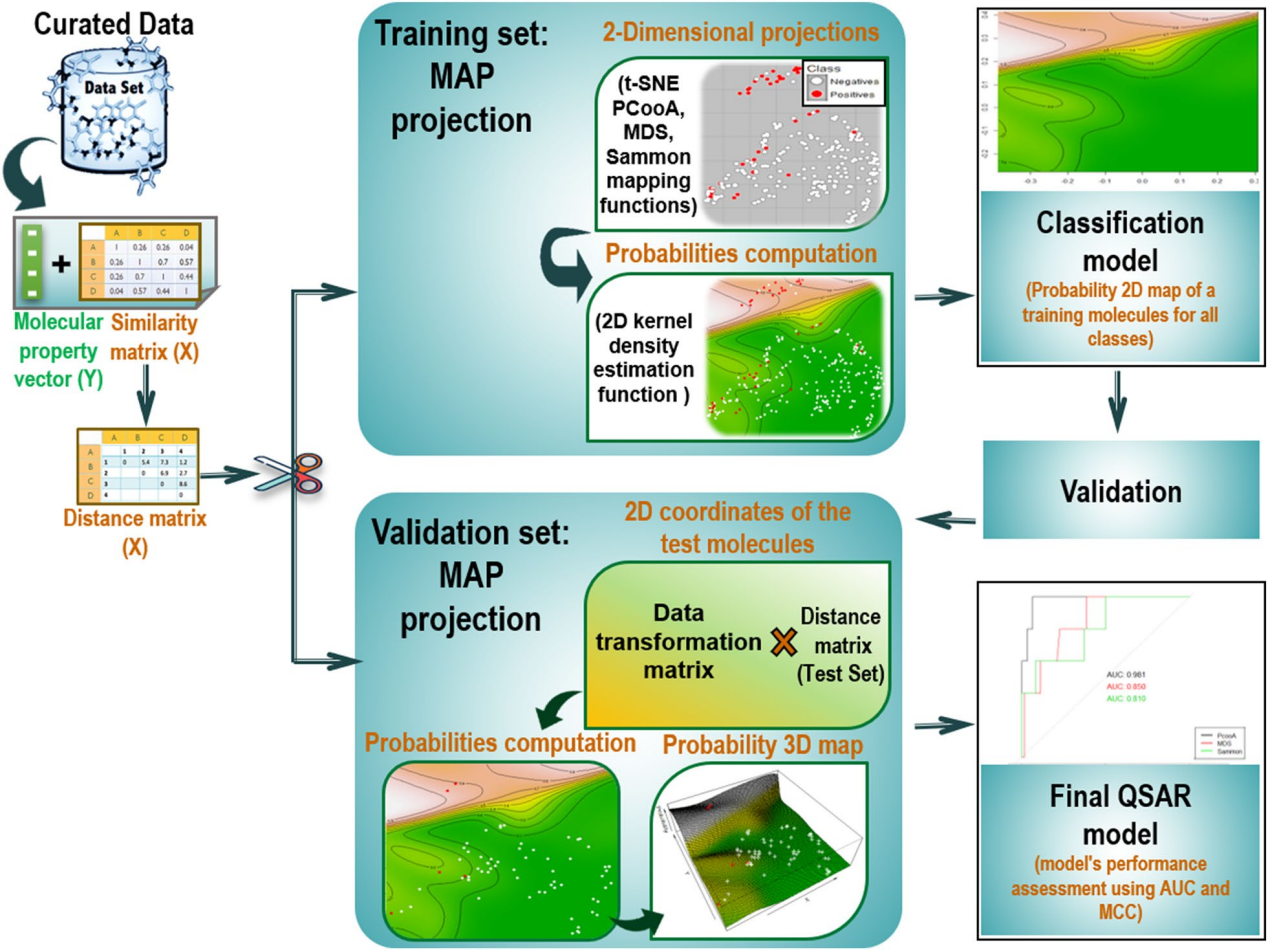
Overview of the methodology

The basic idea of this study is to capture the measured molecular distances according to any proven method and try to represent those molecules in a reduced reference space for analysis and visualization. Many dimensionality reduction methods are extant, [21, 22] and some of the more popular are PCooA, KMDS, SM, and t-SNE [23, 41]. The procedure to create a PSMA can summarily be described in the following steps. First, a full similarity matrix of a molecular data set is computed. Secondly, similarities are transformed into distances and projected into a 2-Dimensional (2D) space using one of the above mapping functions. Finally, the probabilities of this reduced space are computed using a 2D KDE function within a Bayesian perspective [46] to produce a probability map of a projected molecule for all classes. The generated 2D probability map should show the density distribution of training data by mapping the locations of the most likely activity regions of the projected molecular space. Such interactive class probability topographical map (A PSMA) can serve as classification model. To project new molecules into the new reference space, a data transformation matrix can be used for embedding test molecules in the reference activity space. To classify each new projected molecule the generated PSMA is used to calculate their probability of belonging to either class. Models performance was assessed using test molecules predictions (Fig. 1).

### Molecular dis/similarity quantification

Chemical space analysis based on nearest neighbour searches in which molecular similarity analysis is a central task that is based on *Similar Property Principle* [7]. According to this similarity principle, globally similar compounds should have similar properties [9]. Since intermolecular distances between projected molecules are a measure of their molecular similarity or dissimilarity, its quantification must be robust for meaningful spatial/metric space representations, so that they may be able to map similar compounds in contiguous regions, a fundamental aspect for reliable property prediction [6, 7].

For similarity quantification, molecules are translated into numeric data using various molecular representations including structural descriptors and molecular fingerprints [47–49]. Molecular descriptors contain information of structural relevant features of molecules at different levels including constitutional (1D), topological (2D), geometrical (3D) and physico-chemical properties-based (4D) [47, 48]. Molecular fingerprints encode molecular structural information in a bit-string where each bit represents the presence (1) or absence (0) of a structural feature (e.g., chemical substructure, sub-graph, or 2D or 3D pharmacophore). 2D fingerprints are commonly used molecular representations for dis/similarity quantification because comparing bit-string is fast and easy [14, 16, 50–52].

There are some conventional distance metrics like Euclidean, Hamming, Manhattan distance that measure the distance between compounds represented by using descriptors/fingerprints [7, 53]. Some other similarity coefficients are available for binary data (e.g. Tanimoto, Sorensen-Dice, cosine or Tversky) [54, 55]. Of those, the Tanimoto coefficient (Tc) is extensively applied in literature to compute similarity between molecules using molecular fingerprints [53, 56]. Tc compares two fingerprints and counts the number of on-bits (1) common in both with respect to the total number of on-bits (1) in each fingerprint. There are several other approaches that assess similarity using different algorithms based on superposition, molecular graph representations [57] histogram comparisons [58, 59] and Brownian processing of molecules [60]. In this study, we used for molecular similarity assessment a graph matching algorithm, the Non-contiguous Atom Matching Structural similarity (NAMS) [17]. This algorithm uses an atom alignment method to adequately quantify the structural similarity and has a high discriminative power for very similar molecules over other structural or graph matching approaches. NAMS breaks complex molecular structures into simpler parts to reduce molecule to atoms and calculates global structural similarity score from the best alignment between the atoms of compared molecules. NAMS follows an atom matching methodology, which is able to consider the important characteristics of the atoms and bonds such as the chirality and the double bond stereoisomerism. These features are usually ignored in other approaches.

It is important to notice that this approach as it uses a full distance matrix as the basis for its representation, may present some computational challenges over very large data sets. Currently with common lab workstations with 16–32 Gib of RAM, it should be possible to use this methodology with datasets of up to 10,000–15,000 molecules.

### From similarity to distance

As stated above, since molecular similarity is measured by a distance between a pair of molecules in the chosen reference space, a distance function known as metric is mainly required to calculate distances between molecules in metric space representation. A dissimilarity function or a distance function *d*(*x*, *y*) between tho instances *x* and *y* must satisfy the following three basic properties:

- (Property 1) $$d(x, x) = 0$$
- (Property 2) $$d(x, y) \ge 0$$
- (Property 3) $$d(x, z) \le d(x, y) + d(y, z) $$

Which essentially state that a distance between an instance and itself should always be zero, any distance between any instances should never be negative and that the distance between 2 points should respect the triangle inequality. A function that transforms similarity into distance should accordingly be monotonically decreasing and intersect the X-axis precisely at $x=1$. Using these principles similarities and distances can be inter-converted i.e. every similarity metric correspond to a distance metric and vice versa. If similarity function s(x, y) is normalized 0 $\le$ s(x, y) $\le$ 1 and s(x, x) = 1 for all x, y $\in$ X then similarity matrix can be transform into distance matrix with a simple distance functions (see Eqs. 1 [61] and 2 [62])

1 $$\begin{aligned} d(x,y)=\, & {} 1- s(x,y) \end{aligned}$$

2 $$\begin{aligned} s(x,y)=\, & {} \frac{1}{1+d(x,y)} \iff {d(x,y)} = \frac{1}{s(x,y)}-1 \end{aligned}$$

Other complying transformations can also be applied like the negative of the natural logarithm (Eq. 2) [36].

3 $$\begin{aligned} \begin{aligned} d(x,y) = -\ln (s(x,y)) \end{aligned} \end{aligned}$$

These last two equations show the property that similarity values of zero imply an infinite distance, so, for those extreme values, some clamping to a maximum distance may be necessary.

**Fig. 2:**
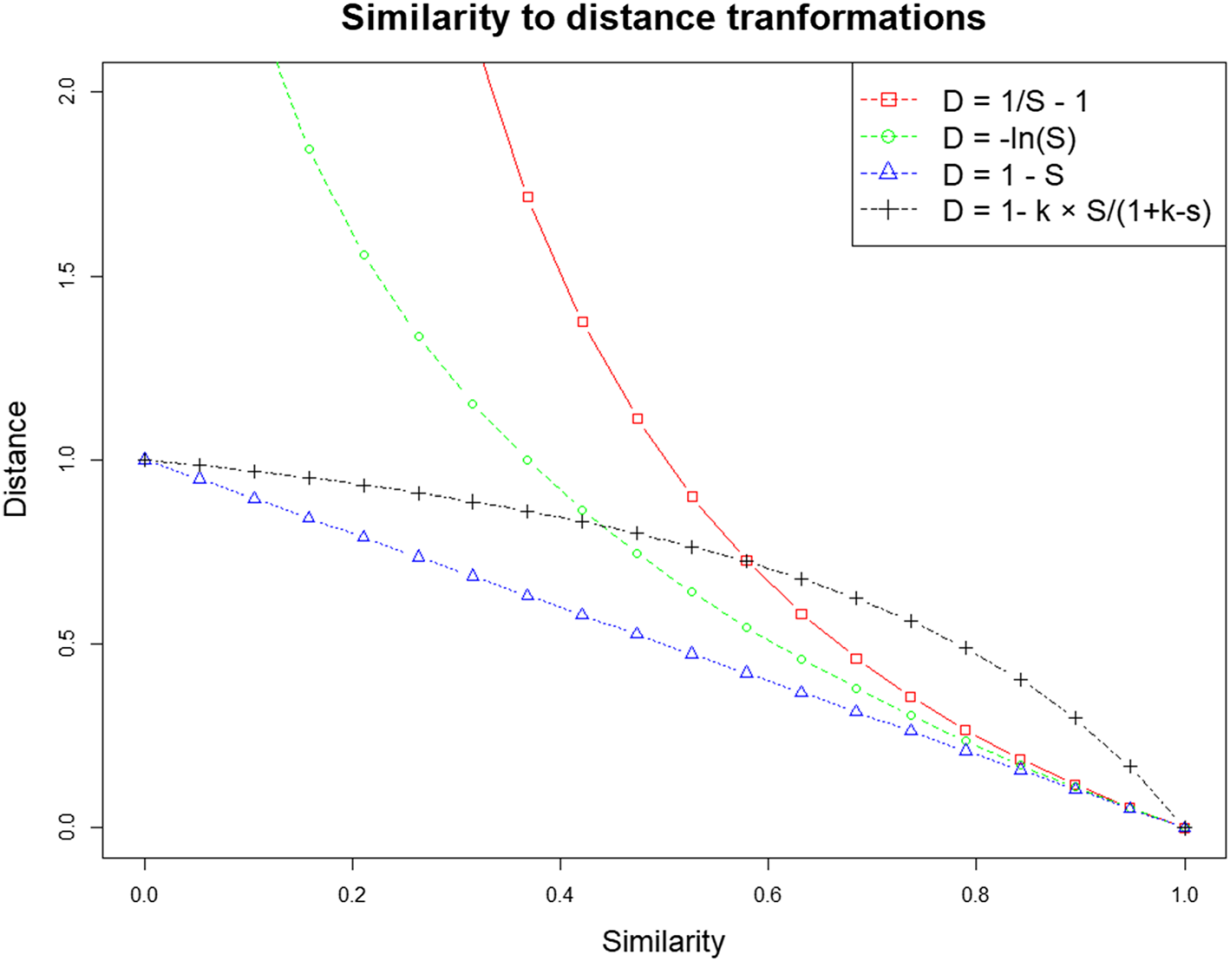
Distance functions for similarity to distance transformations

Within the molecular space a distance function should be modulated to set a particular meaning out of similarity measures. It can be rapidly observed that the last two functions appear concave (Fig. 2), meaning that near the regions that have the lowest similarity, the impact on the resulting distance is the highest, which is counter-intuitive, as typically the conservation of activity for similar molecules is only verified at the highest levels of structural similarity. Such transformation functions may further increase the projection distortion, as most algorithms will tend to minimize the error between the projected distances and the actual distances. A convex curve may solve this problem, by inflating the distances of very similar molecules but, on the other hand, if two molecules are very unrelated, the impact on the transformed distance will appear small. As such we propose the use of the following transformation which uses a parameter *k* that controls the convexity.

4 $$\begin{aligned} \begin{aligned} d(x, y) = 1-\frac{k\times s(x, y)}{1+k- s(x, y)} \end{aligned} \end{aligned}$$

In Eq. 4, small positive values of *k* entail extremely convex functions, while on the other hand, very high values approach $d(x,y)=1-s(x,y)$ (Fig. 2). Empirically and visually we have determined that values of k ranging from 0.3 to 0.5 provide not too abrupt transitions, and a value of 0.382 was used in all problems ($0.382\approx \phi -1$, where $\phi$ is the Golden Ratio)

### Dimensionality reduction

As stated, the visualization of metric space data is a difficult challenge in many different domains of data analysis, as it demands efficient and robust techniques to adequately represent in 2 or 3 dimensions the data variability present in an intrinsic multidimensional problem [21, 22]. The objective of metric space visualization is to generate a topographical map, which should be able to present a visual characterization of molecules by grouping them together on the basis of their structural similarity. As referred, a metric space is an $M\times M$ dimensional distance matrix where *M* compounds are represented each by *M* intermolecular distances. However, it is not trivial to graph the diversity of such high dimensional metric space. As referred, the main objective of dimensionality reduction (DR) in metric spaces is the distance-preservation in original high dimensional space to reduce dimensions. These transformations can be linear or non-linear. The distance-preservation criteria is that any manifold complex geometrical structure of data can be projected into reduced number of dimensions, and the quality of such transformation can be measured by the difference between the original and the projected distances in the new space. A large number of nonlinear DR approaches are available that aim to preserve the local structure of data [21, 22]. In this work we used four of the most widely used DR distance-preserving techniques, namely, Principal Coordinates Analysis (PCooA), Kruskal Multidimensional Scaling (KMDS), Sammon mapping (SM), and t-Distributed Stochastic Neighbor Embedding (t-SNE), for reducing the molecules’ distance matrix in 2D and allow the visualization of the data. After DR, the newly projected instances were divided according to their activity class and a probability function assigned by using a kernel density function to each element of each class.

#### Principal Coordinates Analysis (PCooA)

Principal Coordinates Analysis (PCooA) [27] also known as metric multidimensional scaling (MDS). PCooA relies on a simple generative model possessing all the advantages and drawbacks of Principal components analysis, although its goal is to preserve distances, while PCA aims at preserving the data variance. However, differently from PCA which generally is performed by computing the eigenstructure of the covariance matrix of the data, in PCooA, the basic input is the distance matrix. To the squared of the distance matrix, each element is double centered and, to the resulting matrix, an eigendecomposition is performed. The eigenscaled coordinates of the first *N* eigenvectors are the projected coordinates resulting from this transformation. There are no tuning parameters for PCooA, however, results may vary depending on the distance function used for data metric space representation. It is important to notice that PCooA is only guaranteed to provide an optimal solution if the distances computed between instances are Euclidean, which is clearly not the case. Nonetheless this linear projection into a reduced dimension space has been used in other studies (e.g. [36]). It would have been possible to transform the existing distances into Euclidean distances, but as to make a direct comparison between projection heuristics, it was opted to use the exact same data for all methods without any further processing. Many implementations of KMDS or SM use the results of PCooA transformation as a starting point, which were also used non-transformed.

#### Kruskal Multidimensional Scaling (KMDS)

Non-metric multidimensional scaling was developed by Kruskal [28] for resolving problems related to the linear multidimensional scaling algorithms, like PCooA. The KMDS is based on numerical optimization methods. This method uses ordinal information (i.e., proximity ranks) and then calculates the scaled proximities using monotonic transformation to determine the high-dimensional structure of data set. Finally, to visualize data in low dimensional features space, KMDS finds the best possible projections with minimum squared differences between the initial distances and the scaled ranking of the distances. Thus, in contrast to PCooA, KMDS does not attempt to directly preserve distances between the data points in the initial space but rather its order, or ranking, of the distances between objects [22]. KMDS optimizes the following stress function or error function (Eq. 5) to estimate the preservation of the pairwise distances (goodness of fit).

5 $$\begin{aligned} \begin{aligned} {\displaystyle {\text { Kruskal's stress }}={\sqrt{\frac{\sum _{i,j} (d(i,j) - \widehat{d}(i,j)^2}{\sum _{i,j}d^2(i,j)} }}} \end{aligned} \end{aligned}$$

where *d*(*i*, *j*) are the collected proximities and $\widehat{d}(i,j)$ is the distance measured between the $i{\text {th}}$ and $j{\text {th}}$ objects in low-dimensional representations

#### Sammon mapping

In 1969 Sammon [29] developed a non-linear variant of MDS, which is referred as Sammon mapping, Sammon’s nonlinear mapping and NLM (Non-Linear Mapping). The word “mapping” used to represent the main objective of the method, which was to establish a mapping between a high-dimensional metric space and a lower-dimensional feature space. But, to some extent the ’mapping’ word is misleading as it does not exactly generate a continuous mapping between these two spatial representations. The main goal of Sammon’s algorithm is a dimensionality reduction of a finite set of objects/points by following the same basic principle of MDS algorithm. The main modification is its efficient optimization technique to minimize the Sammon’s stress function (Eq. 6) by calculating its normalized value by the initial space distances. Sammon’s algorithm does not require any parameter optimization, but results may vary depending on the chosen different dissimilarity measures.

6 $${\text{Sammon's}}\,{\text{stress}} = \frac{1}{{\sum\nolimits_{i < j} {d\left( {i,\,j} \right)} }}\sum\limits_{i < j} {\frac{{\left( {d\left( {i,\,j} \right) - \widehat d{{\left( {i,\,j} \right)}^2}} \right)}}{{d\left( {i,\,j} \right)}}} $$

where *d*(*i*, *j*) are orignal distances and $\widehat{d}(i,j)$ are distances between the $i{\text {th}}$ and $j{\text {th}}$ objects in reduced space.

#### t-Distributed Stochastic Neighbor Embedding (t-SNE)

t-Distributed Stochastic Neighbor Embedding (t-SNE) [39] is a variant of Stochastic Neighbor Embedding (SNE) and was developed to solve two basic problems of the SNE algorithm including difficult optimization of a cost function and a problem referred as “crowding problem”. The main objective of both methods SNE and t-SNE is similar to MDS to projected objects in reduced space, such that the pairwise distances between projected objects reflect the original distances between objects as good as possible; although this distance preservation is achieved in a non-linear way. t-SNE algorithm focuses on local data structures, to generate well-separated clusters. One of the key characteristics of this method is that the new distances of objects in the reduced feature space are determined probabilistically with close objects having a much higher probability of staying together in the new space than distant objects. In contrast to SNE, t-SNE does not compute Gaussian “induced” probabilities between each pair of points in embedded space, instead it uses a heavy-tailed Student’s t-distribution for the same purpose so as to avoid the projection of points to the same place (crowding effect). This method consequently allows efficient visualization of moderate distances in the initial space by larger distances in graphical configuration of projected space. Differently from the other methods, t-SNE is a probabilistic approach, thus different runs may produce different maps.

### Probabilities density estimation

The probability density function (PDF) is an informal way to explore and analyse the properties of any given quantitative variable. The PDF gives a natural description of the distribution of any random variable by specifying its probability for all values of its range. Since robust estimation of the probability density can be used to solve regression and classification problems, the PDF is a fundamental concept in data analysis [40, 46]

The PDF for any given variable can be estimated using either parametric methods that assume the density function has a standard distribution function. As an example, if we assume a continuous variable has a normal distribution, then it is possible to compute the full PDF of this variable if the mean and the variance of the data are known and confidently mirror that of the original population. Non-parametric methods, on the other hand, are free of any assumptions and estimate probability density solely from data. One of the most common methods in one dimensional variables is to use a gaussian kernel function applied to each observation, and using the scaled sum of each kernel for each point within the defined range of the data. Non-parametric PDF estimation is an extensive research area in field of data exploration [40, 46]. Most of the existing techniques focus on low dimensional densities estimation (1 to 3D) because uni/bivariate PDF is relatively easy; however investigating PDF of data in higher dimensions (multivariate) is difficult and computational expensive. In lower dimensions histograms can be constructed that generate a non smooth representation of the PDF. But for smooth PDF estimation, the usage of kernel density estimation (KDE) is a common method, used in visual data exploration [40, 46]. The multivariate KDE algorithm has been introduced to deal with high dimensional data with improved accuracy and speed [46, 63]. In our analysis we have used a bivariate KDE applied to the chemical data projected in 2D.

#### 2D kernel density estimation

A kernel density estimation function generates an actual distribution of the data by calculating the probability of each data point in the given data without using any reference point [42] or prior assumptions. Kernel probability density function computes the PDF of the projected 2D space by summing up M-dimensional kernels placed on every projected coordinate. The basic kernel estimator can be expressed as

7 $$\begin{aligned} \begin{aligned} \widehat{f}(x)= \frac{1}{nh}\sum _{j=1}^{n}K\Big (\frac{x-x_j}{h}\Big ) \end{aligned} \end{aligned}$$

where **K** is a fixed kernel and *h* is the calculated bandwidth for sample $x_1, \ldots , x_n$. Commonly available kernel functions are Gaussian (normal), uniform, cosine, triangle, Epanechnikov, quartic (biweight), and tricube (triweight). The bandwidth, *h*, is a smoothing parameter that influences the width of PDF estimates. Choosing a bandwidth is a compromise between very smooth estimates (large *h* values) to remove insignificant bumps and wiggly estimates to find out real peaks (small *h* values). In this study, we applied a two-dimensional KDE with a Gaussian kernel [46] to calculate densities in the two-dimensional reduced space. It is defined as

8 $$\begin{aligned} \begin{aligned} f(x,y)= \frac{\sum _{s}\phi \Big (\frac{(x-x_s)}{h_x}\Big ) \phi \Big (\frac{(y-y_s)}{h_y}\Big ) }{nh_xh_y} \end{aligned} \end{aligned}$$

For determining the bandwidth ($h_n$), we used Silverman’s heuristic approach [40] ($h_n$) for the Gaussian kernel function (Eq. 6) [46].

9 $$\begin{aligned} \begin{aligned} h_n\approx 1.06\min \Big (\widehat{\sigma }, \frac{R}{1.34}\Big )M,^{-\frac{1}{5}} \end{aligned} \end{aligned}$$

where $\widehat{\sigma }$ is the standard deviation of the reference coordinate, *R*, the difference between the 2nd and 3rd quartile and *M* the number of projected points.

In QSAR modelling, KDE is usually explored as an interpolation method to define the applicability domain of generated classification models [45]. Among the most widely used multivariate (high dimensional metric space) interpolation approaches (e.g., range-based, distance-based, geometrical), KDE is considered as one of the more advanced and accurate methods for calculating the applicability domain [42–44]. However, to the best of our knowledge, KDE is not used for visualization nor data classification over 2D activity landscapes.

### Defining active probability regions

In the available literature, several other methods have been used for data visualization of the molecular space. Yet, in all cases each projected point is associated with its measured activity value and surfaces are generated according to the activity magnitude of each molecule or colour codes are used to differentiate different activities [1, 6, 23, 35]. In all these approaches, along with all referred issues in data visualizing methods most implemented interpolation methods are not adequate as classification tools.

To clearly identify the spatial regions where is a higher probability of finding active compounds, to the 2D projected molecules, training data was divided into two classes of active and inactive molecules according to a predefined activity threshold. For both partitions a kernel density map (KDM) is computed, using a common bandwidth, previously computed with all the data. Each KDM can be seen as a measure of the likelihood of a molecule being a negative or a positive depending on its position on the 2D space, as each KDM is an actual probability function, with an integral summing to one. To compute the posteriors of both KDMs it is necessary to accommodate the data priors. Following Bayes’ theorem [64], the posterior probability density (likelihood/probability of a randomly projected new molecule to be in positive class) can be calculated by normalizing the product of the conditional density probability (projected KDM) with the prior probability density of the given partition (positive or negative). Thus to identify whether or not a molecule in (*x*, *y*) coordinates being active it is necessary to evaluate each of both Eqs. (10 and 11)

10 $$\begin{aligned}&P(m_+\mid (x,y) ) = \frac{P((x,y) \mid m_+) P(m_+)}{P((x,y))} \end{aligned}$$

11 $$\begin{aligned}&P(m_- \mid (x,y) ) = \frac{P((x,y) \mid m_-) P(m_-)}{P((x,y))} \end{aligned}$$

where $m_+$ and $m_-$, stand for active (positive) and non-active (negative) molecules. $P((x,y) \mid m_+)$ is the actual value of the KDM of positive molecules (the likelihood of being positive) and $P(m_+)$ the prior probability of the molecule being active. This illustrates that in the end it should be possible to compute the posterior probability $P(m_+\mid (x,y) )$. The corresponding meanings stand for Eq. 11 that quantify the likelihood, prior and posterior probabilities for the inactive molecules

These observations show that it is possible to compute an activity probability surface using the 2D coordinates of the projected molecules. This surface can therefore be visualized and it should be able to capture the more promising activity regions in the chemical landscape. Furthermore as this surface corresponds to an actual activity posterior map, this visualization tool could be used as a classifier, an actual spatial classification model that approximates a 2D Bayes classifier.

### Test set embedding and model validation

The creation of 2D surfaces from the original data will necessarily cause some loss of information. It is thus required to verify if the activity maps constructed are valid in the face of new observations. Therefore to assess model quality, each data set was randomly split into training and test sets. The training set was used to create the model surface and the test set molecules were later embedded, using a linear projection function. If we assume that the original distance matrix is *D* (an *M* $\times$ *M* matrix) that we are going to project into a reduced subspace of *P* dimensions, which we will call *C* (an *M* $\times$ *P* matrix with one row for each instance and *P* columns) corresponding to the molecule coordinates in the new space. As, in the present case, we are transforming into a 2D space, *P* must be equal to 2. We can then assume that there is a transformation *f*() that transforms *D* into *C*: $f(D)=C$. The purpose of any multidimensional scaling algorithm is getting the best *f*() for translating *D* into *C*, according to different stress measures and criteria. A very simple way to model this *f*() function is to assume it as a linear transformation of *D* into *C*. As any linear transformation can be thought as a matrix, which we will call *T*, we can then think of such a transformation as:

12 $$\begin{aligned} \begin{aligned} D\cdot T = C \end{aligned} \end{aligned}$$

The matrix *D* is the original training set distance matrix. After the multidimensional scaling heuristic has been processed we have the projected coordinates of the training set into the new space (*C*). With these, *T* can then be computed by pre-multiplying each part of the equation by the inverse of *D* ($D^{-1}$): Thus

13 $$\begin{aligned} \begin{aligned} D^{-1} \cdot D \cdot T = D^{-1} \cdot C \end{aligned} \end{aligned}$$

Which simplifies into

14 $$\begin{aligned} \begin{aligned} T = D^{-1} \cdot C \end{aligned} \end{aligned}$$

The *T* transformation can then be computed by multiplying the inverse of the training set distance matrix with the matrix of the projected coordinates. This approach is applicable to all projections and, as long as the new instances are close to the training set, this projection should provide an adequate (but not exact) transformation. It is obvious that the projections of the training set Distance matrix(*D*) using *T* will coincide exactly with *C*. To project any new molecule into the new reference space it is only necessary to compute its distances to the molecules of the training set, which will then be transformed by multiplying the transpose of that vector with matrix *T*.

As the projected coordinates of each molecule into the new reference space, it should be easy to compute its activity probability (Eqs. 10 and 11). As the result is a probability function, the model’s performance was assessed using AUC, which measures the entire two-dimensional area underneath the entire receiver operating characteristic (ROC) curve created by plotting the sensitivity/recall/true positive rate (TPR) against the false positive rate (FPR) (Eqs. 15 and 16).

15 $$\begin{aligned}&\text {Sensitivity} = \frac{TP}{TP+FN} \end{aligned}$$

16 $$\begin{aligned}&FPR= 1 -\text {Specificity} = 1 - \frac{TN}{TN+FP} \end{aligned}$$

where, for both eqs., TP are the true positives, TN, the true negatives, FP the false positives and FN, the false negatives. For AUC computation, the positive accepting threshold is changed, and thus the values of these quantities will change accordingly. and provide the data for building the ROC curve.

A second, more stringent criterion is the use of the Matthews Correlation Criterion (MCC) [65], which encompasses the quantities defined above into one statistic that has been widely used for assessing the quality of binary classification models (Eq. 17). Differently from the AUC, the MCC will consider as positives only the instances where $P(M_+\mid (x,y) > P(M_-\mid (x,y) ))$

17 $$\begin{aligned} \begin{aligned} {\displaystyle {\text {MCC}}={\frac{TP\times TN-FP\times FN}{\sqrt{(TP+FP)(TP+FN)(TN+FP)(TN+FN)}}}} \end{aligned} \end{aligned}$$

## Data

The designed methodology was tested over four human protein targets (Table 1), retrieved from ChEMBL23 [66]. We have looked for data sets for which biological activity was measured as $K_i$ as it quantifies a ligand-receptor interaction based on the equilibrium dissociation constant (*K*) where smaller value corresponds higher ligand-receptor binding affinities and vice versa [67]. The selected data sets were curated using an automated QSAR modelling workflow [68] which essentially removed duplicates and curated the structures by removing salt groups and complex compounds. Of the four data sets selected, only was further trimmed, as we were looking for a data set where the experimental conditions were similar. As such, for the Sigma 1 receptor only molecules for which $K_i$ resulted from the displacement of [3H]-pentazocine were considered. All data sets were divided into two classes using a cut-off activity value to discriminate highly active molecules ($K_i$ $\le$ 10.0 nM) as positives and less active and non-active molecules ($K_i$ > 10.0 nM) as negatives. This low cut-off value was selected to counter the positive bias in public repositories such as ChEMBL, which is an unrealistic assumption in actual drug development scenarios, where the active/non-active ratio is typically much lower. Such stringent threshold resulted in purposely unbalanced data sets with a much larger number of negatives than positives, which is a known characteristic of most problem sets in real world in-silico drug development. Such unbalance is an hindrance in model learning, and makes the fitting process typically much harder.

**Table 1:** Data set description—data set sizes discriminated by positives and negatives within training/testing data split

Target protein name	Uniprot ID	Training set		Test set		Mean distance	Distance std. dev.
		Positives	Negatives	Positives	Negatives		
Sigma non-opioid intracellular receptor 1 (SIGMAR1)	Q99720	46	135	10	35	0.79	0.13
Histamine H1 receptor (HRH1)	P35367	184	783	46	195	0.83	0.08
Potassium voltage-gated channel subfamily H member 2 (HERG)	Q12809	39	1142	12	283	0.84	0.06
D(1B) dopamine receptor (DRD5)	P21918	41	231	5	62	0.80	0.10

Includes the average computational distance between compounds of each data set and its respective standard deviation

To assess the structural diversity of of each data set the average distance between all pairs of molecules was computed. For all data sets the average distance was about 0.8, with a low standard deviation $(\sim 0.09)$ which indicates that these data sets are very diverse with a large majority of molecules with virtually no ascertainable similarity to most of the others in each data set.

## Implementation

All analysis was implemented using R software (version 3.4.4) [69] on a PC desktop with a Core i7 Processor (3.41 GHz) and 16 GB RAM. Data sets of all selected problems were divided into a training set and test set using a random partition with (20/80)% ratio. Similarity matrices (*M* $\times$ *M* matrix containing intermolecular similarities) of all data sets were computed using NAMS [17] that allows for the computation of pairwise similarities between all molecules within a database. All the other NAMS parameters were left as default. Similarity matrices were converted into dissimilarity matrices (metric space representation) using Eq. 4, with $k = 0.382$ for all data sets. For the DR processes, R cmdscale function [70] was used for PCooA, two functions from R package MASS [46] including isoMDS and sammon was used for KMDS and SM respectively. We used the t-SNE implementation from R library Rtsne [39]. Finally, for computing the kernel desnity map in 2D, the kde2d function from R package MASS [46] was used. The bandwidths for the positive and negative maps were calculated beforehand using the bandwidth.nrd function (see Eq. 9).

## Results and discussion

**Fig. 3:**
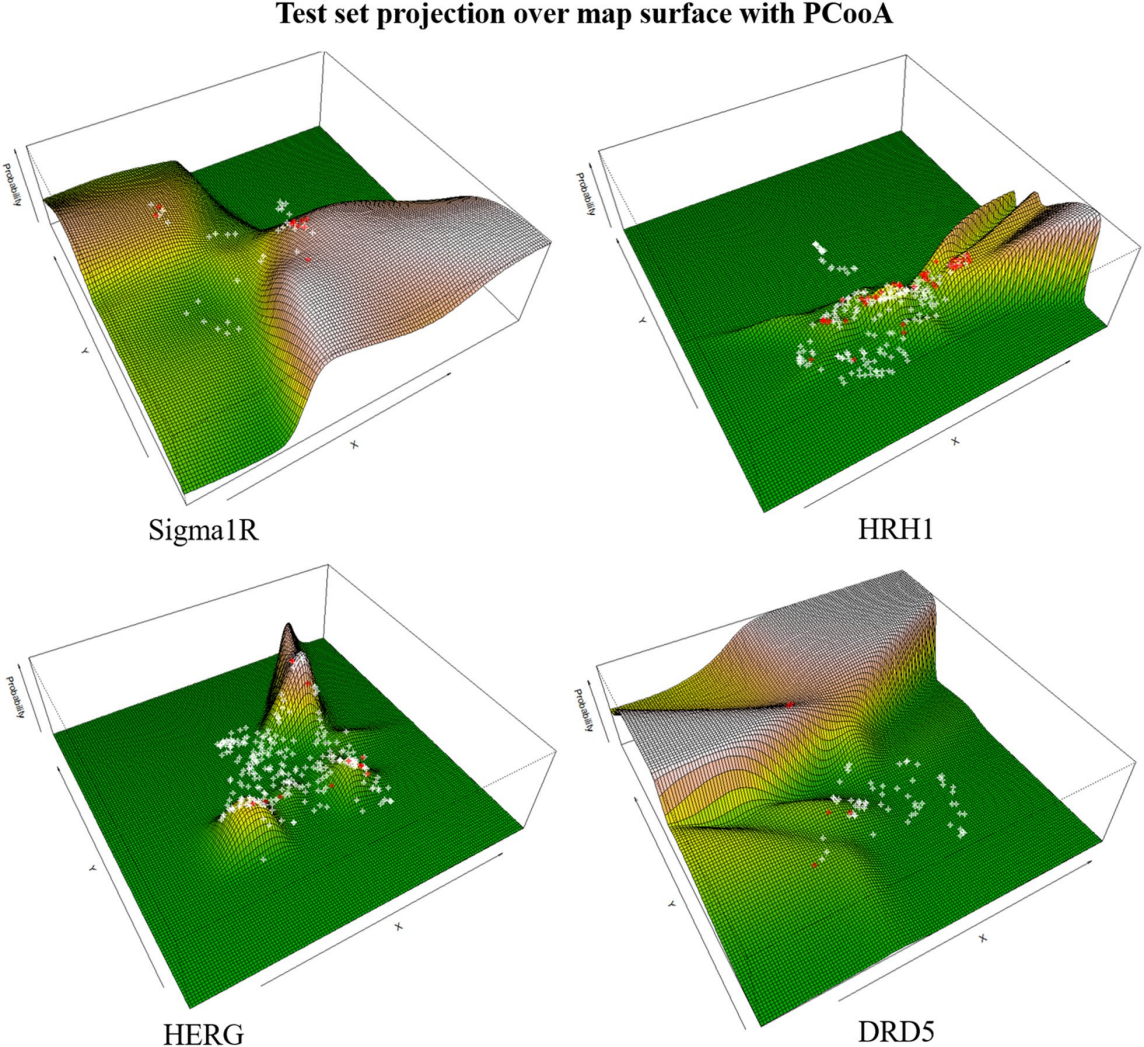
Test set projection over map surface (PSMA) with PCooA. Surfaces represents higher probability locations. red – circles are ground truth positives, white are ground truth negatives

We generated the activity ($K_i$) probability maps (PSMA) for four different problems (*SIGMAR1, HRH1, HERG,* and *DRD5*) using 4 different DR methods: PCooa, KMDS, SM, and t-SNE. For each we have produced the probability of activity surface maps. These PSMAs typically show consistency, and the regions with the highest probability of activity appear most of the times well differentiated from the negative regions. Figure 3 shows the results of PCooA projection for the 4 data sets, computed solely from the 80% training data. The PSMA for all projection functions appears on supplementary material (Additional file 1: Figure S1a, S1b, S1c). In these probability maps, surface height mirrors the kernel density distribution of active molecules (positive class) and the colour represents higher probability locations (most likely activity regions). It is interesting to verify that the visual complexity and asymmetry of the resulting maps clearly shows that a naive-Bayes approach would be inadequate for this type of modeling, where the data clustering patterns make obvious the interdependence of the data in the projection axes.

**Fig. 4:**
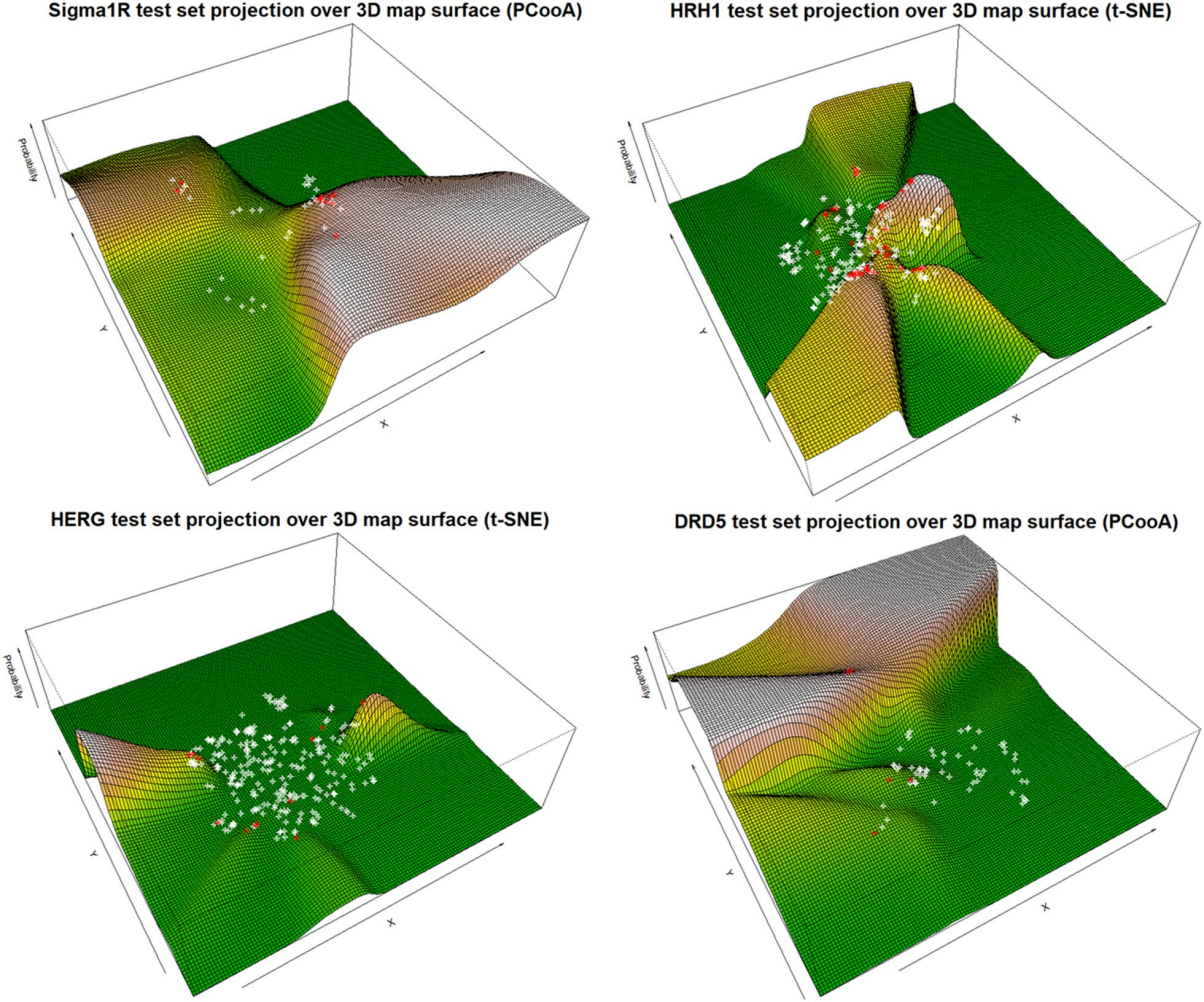
Test set projection over map surface of selected PSMAs with highest performance. Surfaces represents higher probability locations. red-circles are ground truth positives, white are ground truth negatives

To check the quality of the produced probability surface maps, the test set molecules were projected into the new reference plane. The performance of all sixteen generated PSMAs was assessed using AUC and MCC. AUC testing results range from 0.77 to 0.98 and MCC score ranges between 0.18 to 0.77 (Table 2). In two data sets (*SIGMAR1* and *DRD5*), PCooA performance was better than the other DR methods while for *HRH1* and *HERG* t-SNE outperformed the others. All DR approaches provided generally good AUC results. The overall performance of PCooA and t-SNE was roughly the same (average AUC = 0.86) in all four problems with a slightly (and not statistically significant) more positive outcome for PCooA with the MCC score. The test set projections over the best PSMAs for each data set shows ground truth active molecules (as red circles) typically within the highest probability of activity regions (Fig. 4). In the present analysis is several cases, the MCC was low, albeit always showing clear discriminant power. On the other hand, the AUC score was consistently high.

**Table 2:** Results on validation set

Target protein name	PCooA		KMDS		SM		t-SNE	
	AUC	MCC	AUC	MCC	AUC	MCC	AUC	MCC
Sigma non-opioid intracellular receptor 1 (Sigma1R)	0.87(*)	0.63	0.80	0.60	0.79	0.55	0.79	0.47
Histamine H1 receptor (HRH1)	0.80	0.45	0.83	0.43	0.78	0.36	0.87(*)	0.54
Potassium voltage-gated channel subfamily H member 2 (HERG)	0.80	0.18	0.77	0.24	0.80	0.25	0.89(*)	0.33
D(1B) dopamine receptor (DRD5)	0.98(*)	0.77	0.86	0.32	0.80	0.42	0.90	0.41
Overall performance (average score)	0.86	0.51	0.82	0.40	0.79	0.40	0.86	0.44

*PCooA* Principal co-ordinates analysis,* KMDS* Kruskal Multidimensional Scaling,* SM* Sammon mapping,* t-SNE* t-distributed stochastic neighbor em-bedding

(*)—best model

**Fig. 5:**
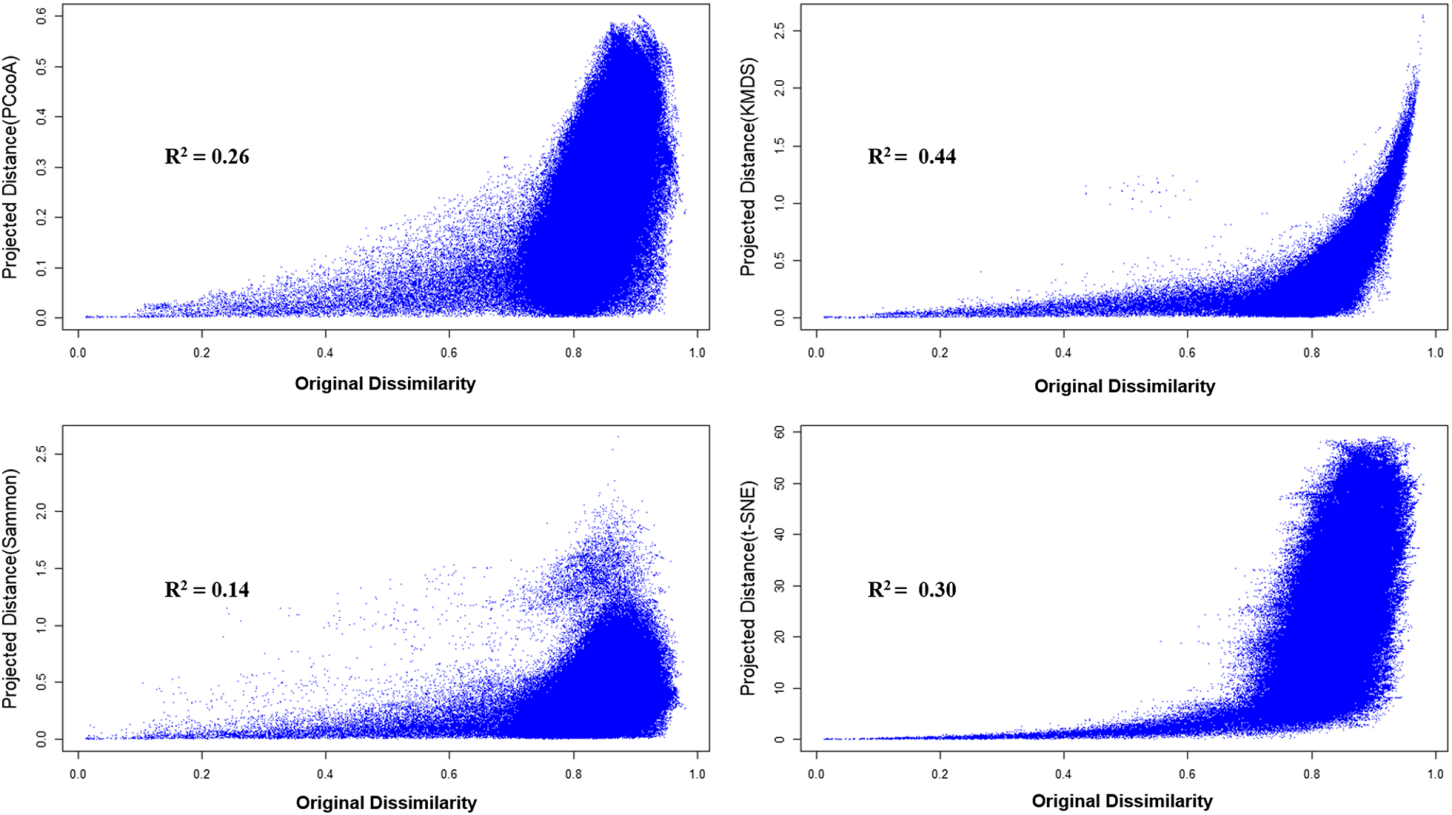
HERG shepard plot for PCooA, KMDS, SM and t-SNE

Since dimensionality reduction is one of the important task in data visualization where it is really necessary to capture the maximum original data information in the new reduced space, Shepard plots [46] were generated to analyze how much molecular initial proximity relationship remained intact. In Shepard plots the original distances are plotted against the projected distances and, ideally, the points (both distances) should lie on a straight line, which would indicate zero distortion in the projection function. The Shepard plot for the hERG data set, for all projection functions is shown (Fig. 5). The remaining three data set Shepard plots can be seen in supplementary material (Additional file 1: Figures S2a–c). The 2D projections, for all approaches, showed a similar pattern, in which it can be seen that many large distances in the initial space fail to maintain that separation in the projected space, however, in all cases, very close molecules will always appear close, which shows that locality factors were preserved in all projections, which contributes to explain the quality of the classification models. The projection of dissimilar molecules in the vicinity of similar molecules can generate noise in visualization of the real pattern of the data distribution. This is probably the cause for having low MCC scores in some data sets.

To verify whether the quality of the projection influences the classification results, the $R^2$ coefficient that measures how the projected distances measure against the original distances was calculated (Fig. 5 and Additional file 1: Figure S2). It is apparent that KMDS shows the highest scores while Sammon mapping features the lowest values for all 4 test cases. It is therefore striking that KMDS although always able to produce consistently good models was never the projection that yielded the best results. This may suggest that, on this reduced dimension space, other factors rather than stricter distance preservation may be relevant for accurate model building, and the nonlinear optimization performed by KMDS actually hampers the projection quality for classification purposes.

**Fig. 6:**
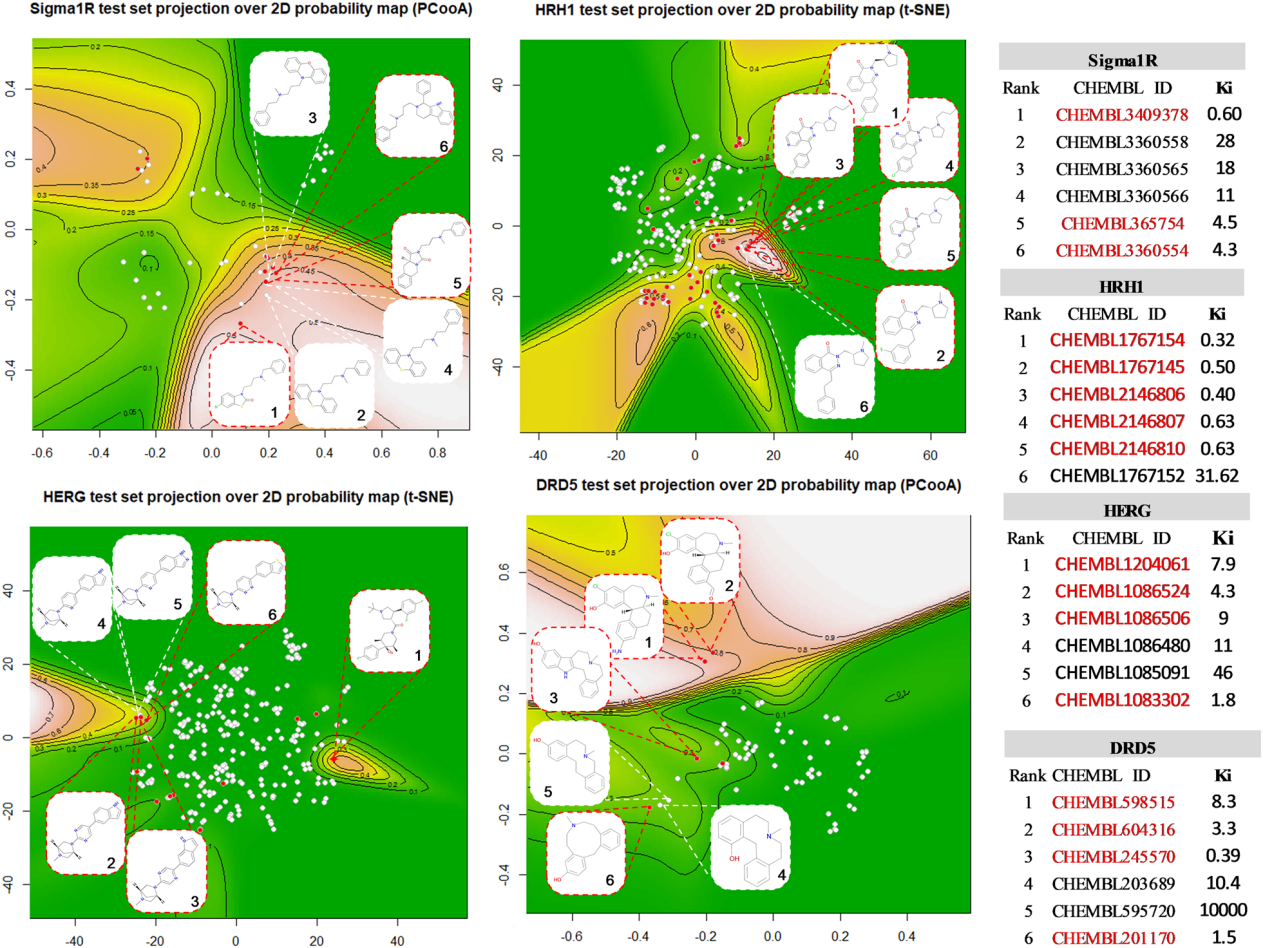
Test set projection over 2D probability map of selected models with highest performance. Contour lines represent 2D kernel density distribution of active molecules (positive class) and the colour other than green represents higher probability locations. Red-circles are ground truth positives, white are ground truth negatives. ChEMBL IDs. in red color text (2D structures within red lined box) are true positives and other are false positives (2D structures within white lined box)

To have a more detailed appraisal of the quality of the test set projections, the 2D molecular structures of top 6 test molecules with higher probability of being actives (predicted positives) are shown within the 2D probability map of the best 2D projections for each data set (Table 2.), along with their ChEMBL IDs (Fig. 6). It can be seen that, with only one exception, in all 4 data sets, all molecules were strong actives, although some not within the strict activity criterion ($K_i\le 10 nM$). For the Sigma 1 Receptor, there were 3 correctly predicted positives, and the three negatives incorrectly predicted had in fact very low $K_i$ values, all of them below 30 nM. For the Histamine 1 receptor, the 5 more likely molecules to be active were all correctly predicted as positives, which is striking as this data set is one of the hardest, with low classification results. The only miss is one molecule (CHEMBL1767152) with a $K_i= 31.62 nM$, therefore with strong activity as well. The humean hERG is the hardest problem, as the number of negative molecules largely outnumbers the positives, nonetheless for the 6 molecules with highest probability, 4 were correctly predicted only two were misses. As in the previous cases, both molecules (CHEMBL1086480 and CHEMBL1085091) are also strong actives, with $K_i \le 50 nM$. The last test set (Dopamine 5 receptor), is the one with the more striking situation, as this was the data set that had the highest classification performance. The two misses, the first molecule had a $K_i=10.4 nM$, thus clearly a borderline molecule. The compound CHEMBL595720, was the only one that on ChEMBL was a clear inactive with a measured $K_i \ge 10,000 nM$. It can be pointed out that, on this specific problem, that molecule is outside the most active region which appears clearly marked on the upper region of the map, with an activity cliff crossing the full surface, identifying the most promising region for finding very active molecules.

## Conclusion

This study aimed initially at presenting a visualization method that is able to capture the highest probability regions for molecules being active. To reach this goal, the molecular spaces of four data sets, captured as similarity matrices that were computed using NAMS, a graph matching algorithm. In a previous study NAMS-based molecular metric space representation was found a reliable approach to establish molecular similarity-activity relationship in QSAR modeling [38]. Accordingly NAMS-based molecular spaces for the selected data sets were reduced into a new reference space in 2D using four different algorithms. The X, Y coordinates generated from each DR methods were used by a 2D kernel density function to generate their corresponding activity probability maps (PSMAs). These PSMAs were able to depict the most likely activity regions, and appear consistent, with active molecules clearly grouping together. The analysis of the produced PSMAs from the 4 data sets showed the reliability of the proposed methodology as it can efficiently produce visual cues as to where the more promising regions of the molecular space are located. The presented approach allows for the projection of new molecules into the new projected space, thus allowing for model assessment with external data. Accordingly, to validate the quality of this 2D representation as a classification model, independent validation sets were projected over the generated PSMAs, and the results were consistently good with AUC values, for the highest scoring projections, ranging from 0.87 to 0.98 and MCC scores ranging from 0.33 to 0.77. Although the followed approach did not aim at optimizing models for getting high classification accuracies, these results are strongly suggestive that it actually is capturing a large part of the modelable aspects of these SAR problems. This approach therefore uses only the 2D structural similarity between molecules to produce a non-parametric model that is both visually informative and shows demonstrable quality as a classification model.

The predictability of the presented spatial classification model (PSMA) is thus an attractive feature for virtual screening using only structural similarity of molecules. The applicability domain of such visual approaches can be vastly increased using larger data sets for any single or multiple targets. Comparatively to traditional QSAR models with a limited applicability domain, this activity space visualization directly uses structural similarity and thus may enhance SAR visualization within large activity spaces.

## Availability and requirements

All data sets and R source code (PSMA.Rmd and PSMA.html) for analysis and inference of molecular activity spaces is available in supplementary material (Additional file 2).

## Supplementary information

**Additional file 1: Figure S1a.** Test set projection over PSMA with KMDS. **b** Test set projection over PSMA with SM. **c** Test set projection over PSMA with t-SNE. **Figure S2a.** DRD5 shepard plot for PCooA, KMDS, SM and t-SNE. **b** HRH1 shepard plot for PCooA, KMDS, SM and t-SNE. **c** SIGMAR1 shepard plot for PCooA, KMDS, SM and t-SNE.

**Additional file 2.** Contains all data sets (SIGMAR1, HRH1, HERG,and DRD5) retrieved from ChEMBL23, their computed NAMS similarities and R source code for analysis and inference of molecular activity spaces.
