# Supplementary figures and images for: A visual approach for analysis and inference of molecular activity spaces
Source: J Cheminform. 2019 Oct 22;11:63. doi: 10.1186/s13321-019-0386-z (PMC6805449; doi:10.1186/s13321-019-0386-z)

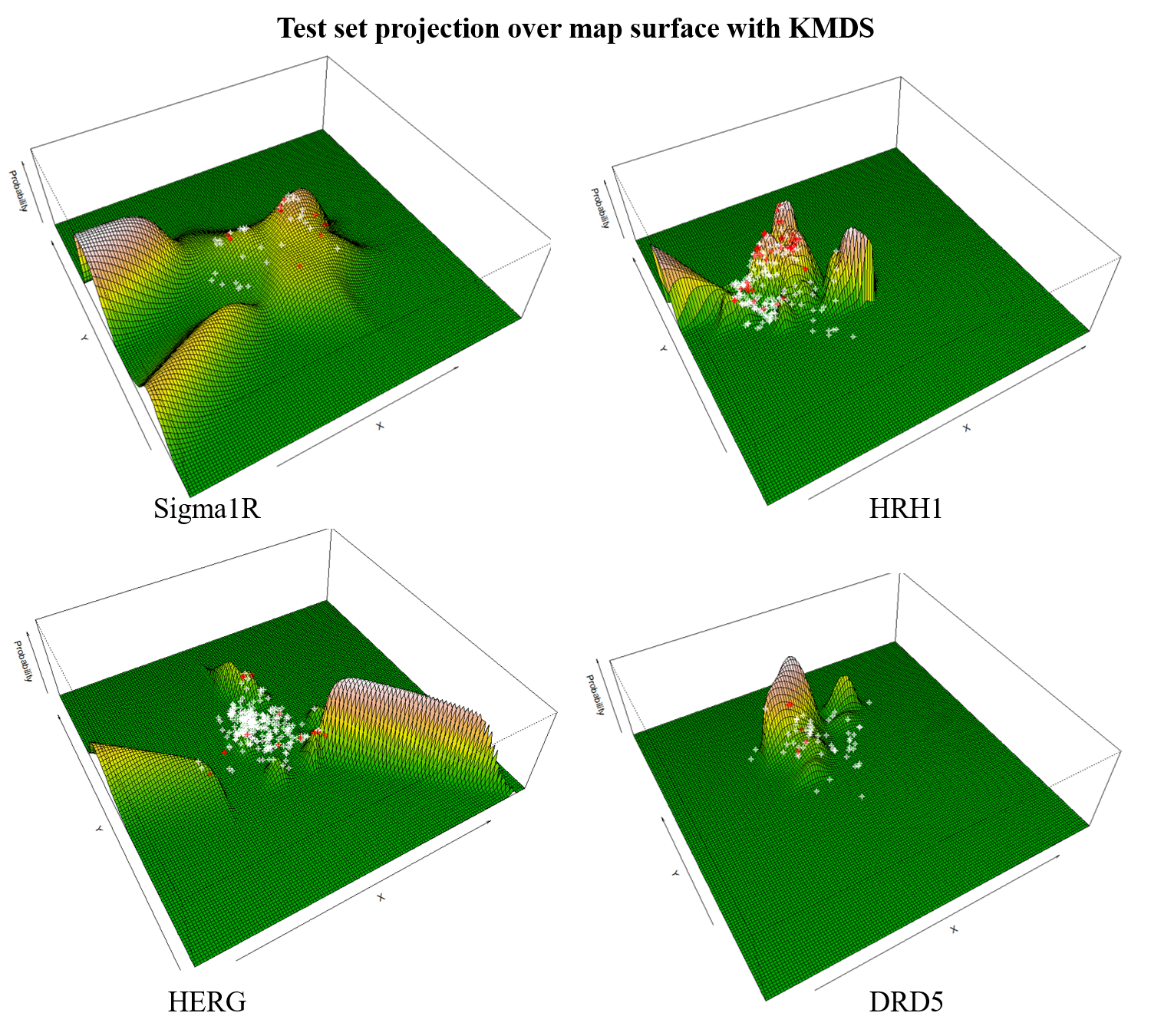

Supplement: Supplementary file 1 — Additional file 1: Figure S1a. Test set projection over PSMA with KMDS. b Test set projection over PSMA with SM. c Test set projection over PSMA with t-SNE. Figure S2a. DRD5 shepard plot for PCooA, KMDS, SM and t-SNE. b HRH1 shepard plot for PCooA, KMDS, SM and t-SNE. c SIGMAR1 shepard plot for PCooA, KMDS, SM and t-SNE. [file 13321_2019_386_MOESM1_ESM.zip › Additional File 1/Figure S1a.png]

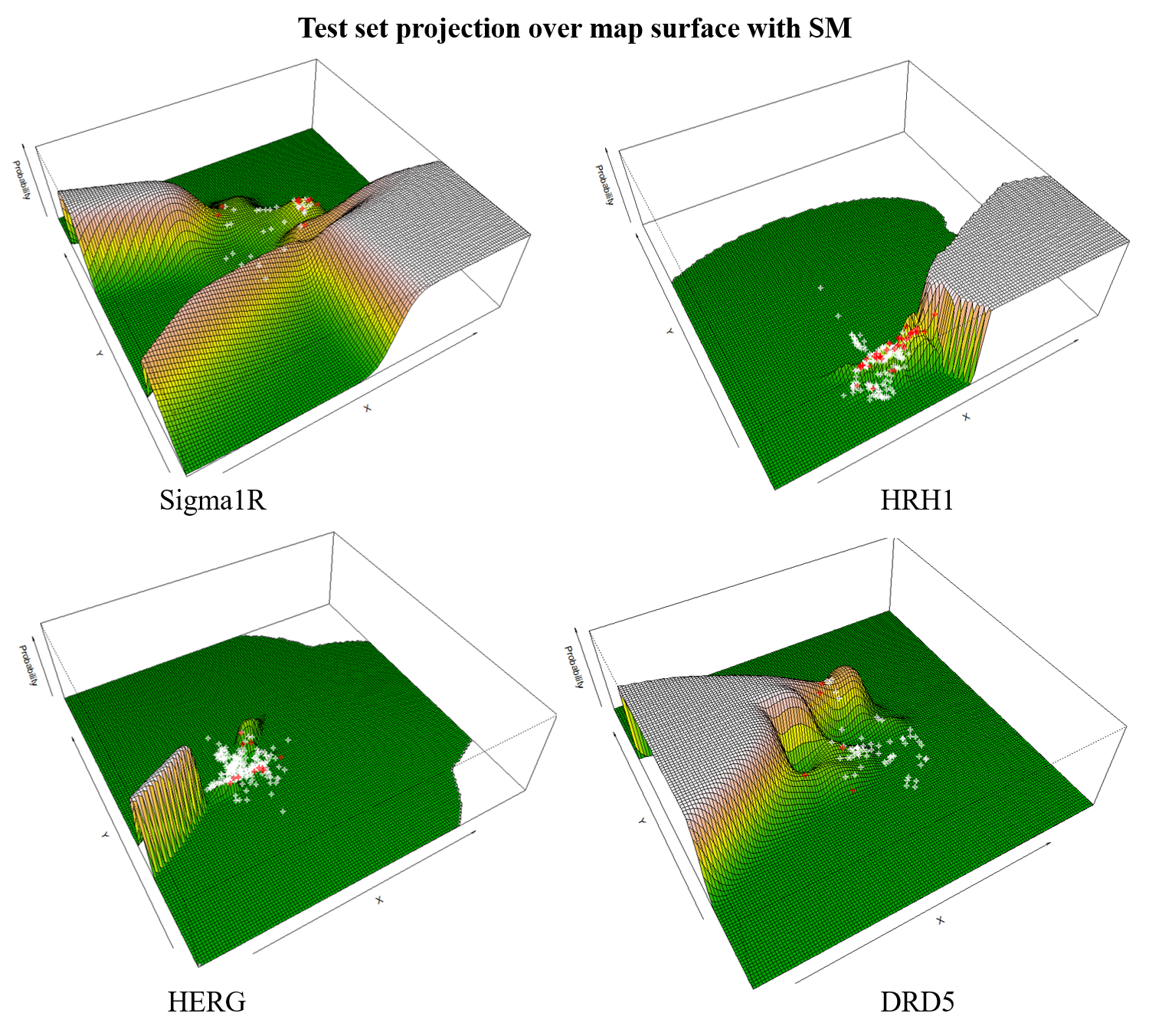

Supplement: Supplementary file 1 — Additional file 1: Figure S1a. Test set projection over PSMA with KMDS. b Test set projection over PSMA with SM. c Test set projection over PSMA with t-SNE. Figure S2a. DRD5 shepard plot for PCooA, KMDS, SM and t-SNE. b HRH1 shepard plot for PCooA, KMDS, SM and t-SNE. c SIGMAR1 shepard plot for PCooA, KMDS, SM and t-SNE. [file 13321_2019_386_MOESM1_ESM.zip › Additional File 1/Figure S1b.png]

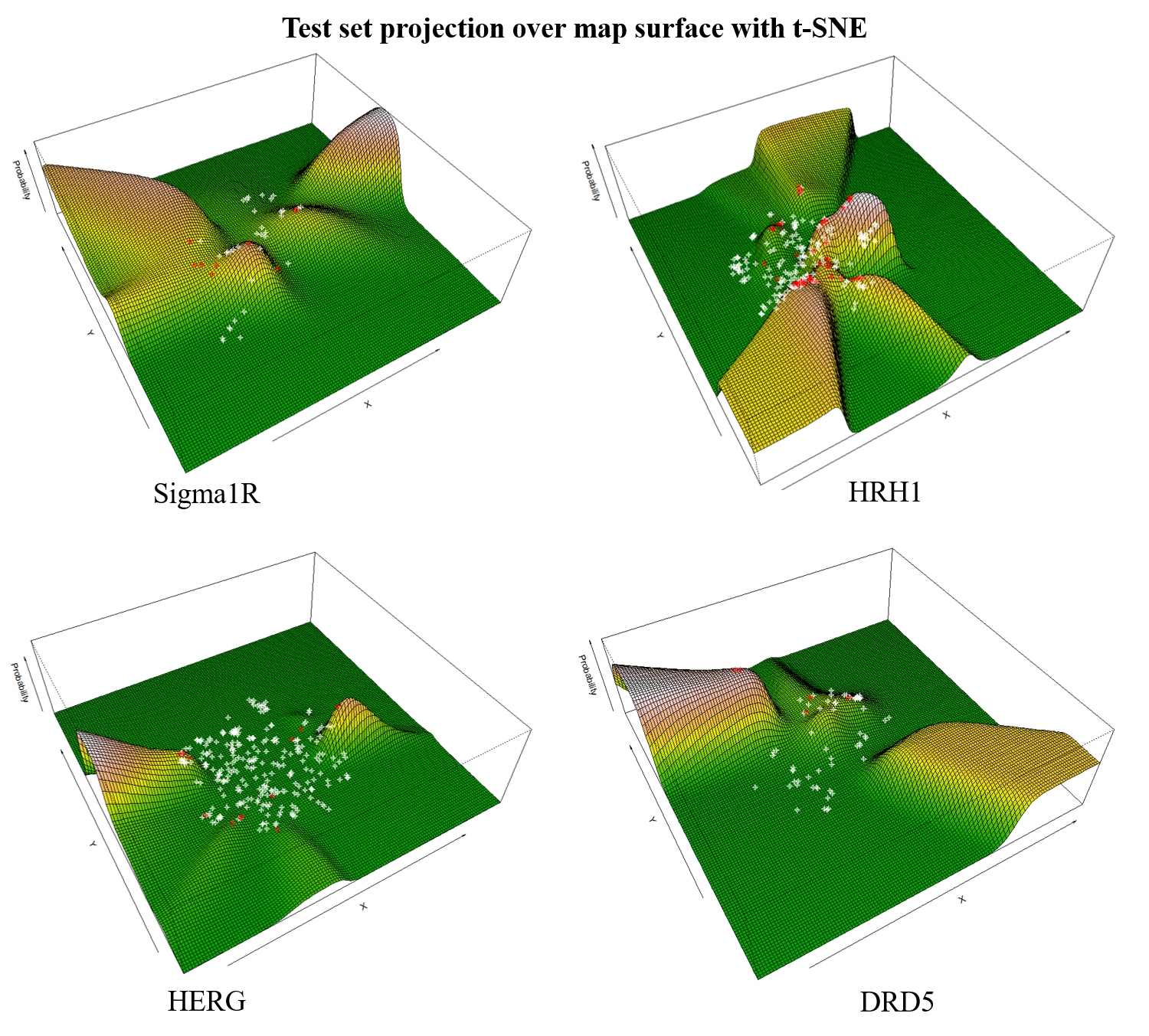

Supplement: Supplementary file 1 — Additional file 1: Figure S1a. Test set projection over PSMA with KMDS. b Test set projection over PSMA with SM. c Test set projection over PSMA with t-SNE. Figure S2a. DRD5 shepard plot for PCooA, KMDS, SM and t-SNE. b HRH1 shepard plot for PCooA, KMDS, SM and t-SNE. c SIGMAR1 shepard plot for PCooA, KMDS, SM and t-SNE. [file 13321_2019_386_MOESM1_ESM.zip › Additional File 1/Figure S1c.png]

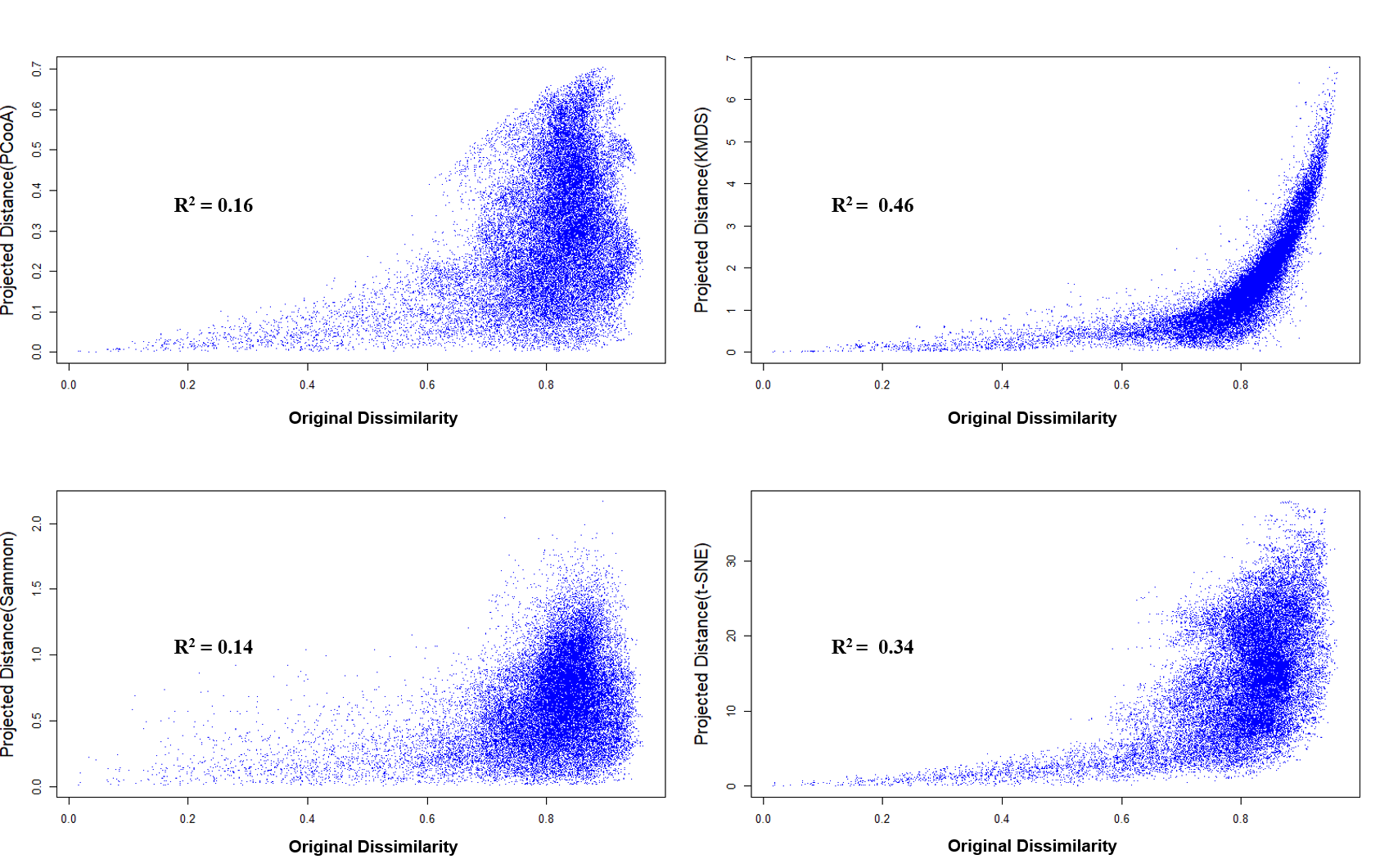

Supplement: Supplementary file 1 — Additional file 1: Figure S1a. Test set projection over PSMA with KMDS. b Test set projection over PSMA with SM. c Test set projection over PSMA with t-SNE. Figure S2a. DRD5 shepard plot for PCooA, KMDS, SM and t-SNE. b HRH1 shepard plot for PCooA, KMDS, SM and t-SNE. c SIGMAR1 shepard plot for PCooA, KMDS, SM and t-SNE. [file 13321_2019_386_MOESM1_ESM.zip › Additional File 1/Figure S2a.png]

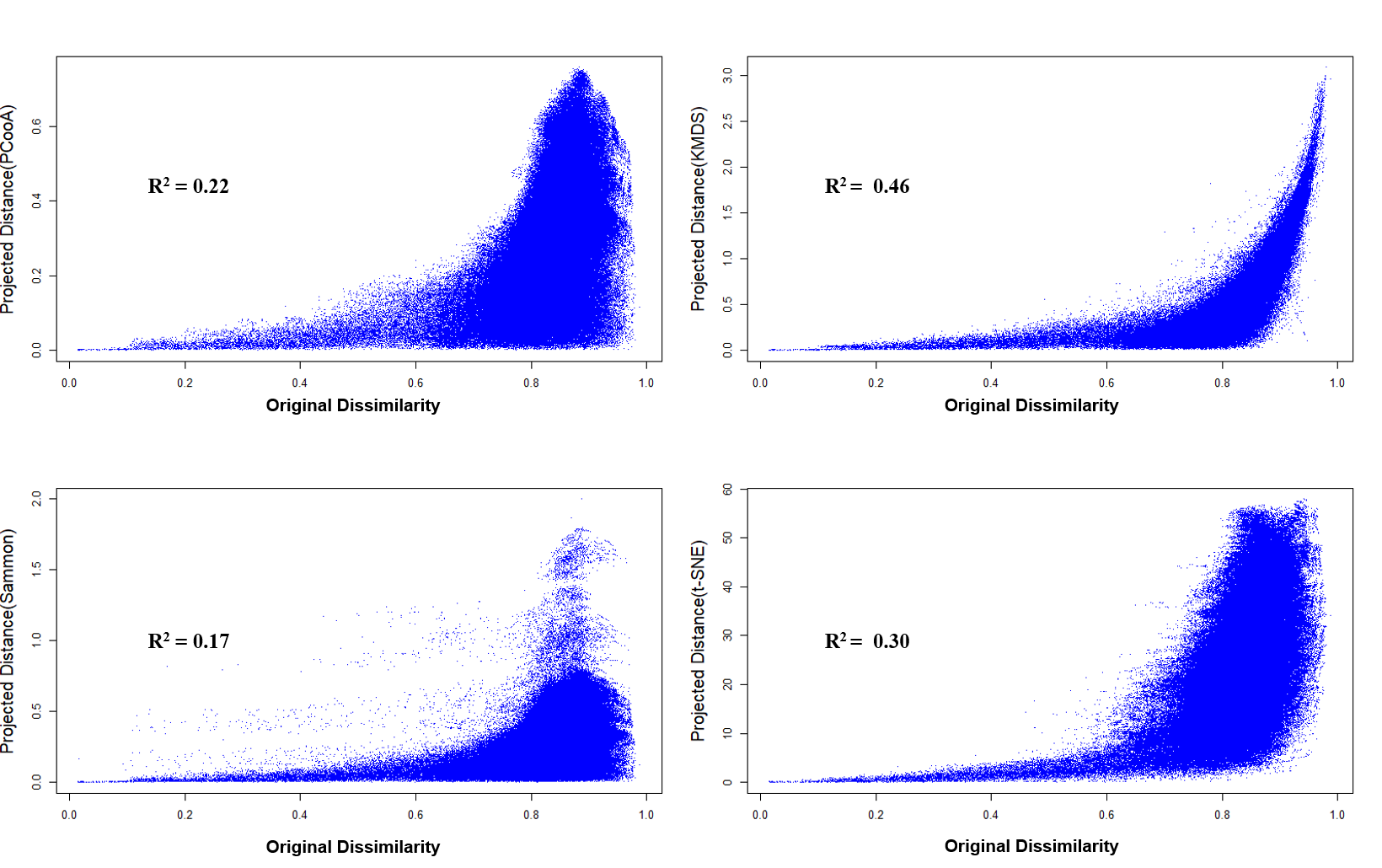

Supplement: Supplementary file 1 — Additional file 1: Figure S1a. Test set projection over PSMA with KMDS. b Test set projection over PSMA with SM. c Test set projection over PSMA with t-SNE. Figure S2a. DRD5 shepard plot for PCooA, KMDS, SM and t-SNE. b HRH1 shepard plot for PCooA, KMDS, SM and t-SNE. c SIGMAR1 shepard plot for PCooA, KMDS, SM and t-SNE. [file 13321_2019_386_MOESM1_ESM.zip › Additional File 1/Figure S2b.png]

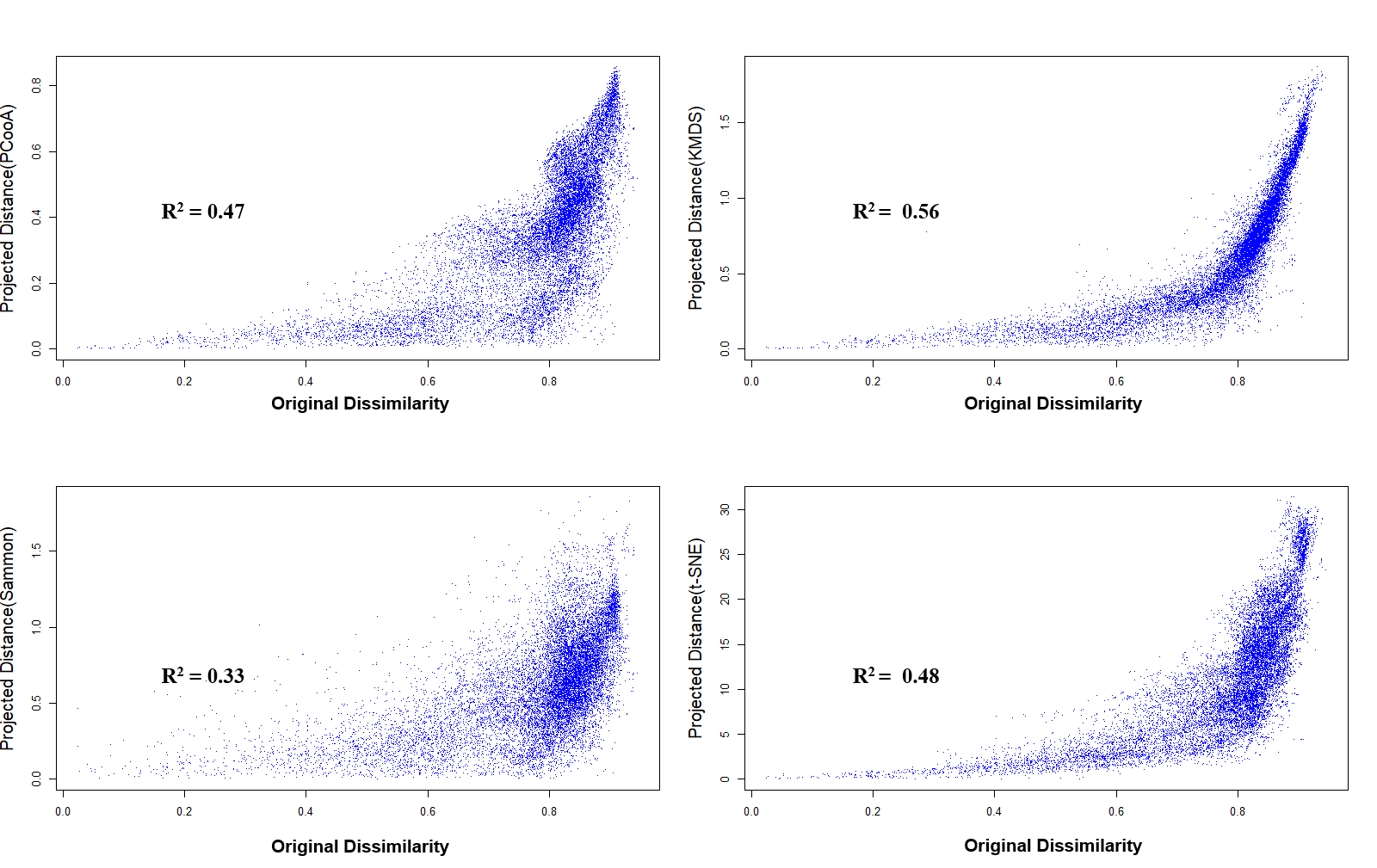

Supplement: Supplementary file 1 — Additional file 1: Figure S1a. Test set projection over PSMA with KMDS. b Test set projection over PSMA with SM. c Test set projection over PSMA with t-SNE. Figure S2a. DRD5 shepard plot for PCooA, KMDS, SM and t-SNE. b HRH1 shepard plot for PCooA, KMDS, SM and t-SNE. c SIGMAR1 shepard plot for PCooA, KMDS, SM and t-SNE. [file 13321_2019_386_MOESM1_ESM.zip › Additional File 1/Figure S2c.png]
