# Supplementary material for: A visual approach for analysis and inference of molecular activity spaces
Source: J Cheminform. 2019 Oct 22;11:63. doi: 10.1186/s13321-019-0386-z (PMC6805449; doi:10.1186/s13321-019-0386-z)
Supplement: Supplementary file 2 — Additional file 2. Contains all data sets (SIGMAR1, HRH1, HERG,and DRD5) retrieved from ChEMBL23, their computed NAMS similarities and R source code for analysis and inference of molecular activity spaces. [file 13321_2019_386_MOESM2_ESM.zip › Additional File 2/PSMA.html]

A visual approach for analysis and inference of molecular activity spaces


# A visual approach for analysis and inference of molecular activity spaces

This pipeline can efficiently be used to build probabilistic surfaces of molecular activity (PSMAs) for visual characterization of molecules in molecular activity spaces. This approach allows building of a non-parametric classification model out of raw similarity data.

The following libraries are required:

```
library(MASS)
library(Rtsne)
library(pROC)
library(mltools)
```

## Methodology

The basic idea of this approach is to capture the measured molecular distances according to any proven method and try to represent those molecules in a reduced reference space for analysis and visualization. The procedure to create a PSMA can be divided into the following steps:

- Full similarity matrix of a molecular data set is computed and transformed into distance
- Metric space is projected into a 2-Dimensional (2D) space using dimension reduction algorithms (PCooA, Kruskal multidimensional scaling, Sammon mapping and t-SNE)
- Finally, the probabilities of the reduced space are computed using a 2D KDE function within a Bayesian perspective to produce a probability map of a projected molecule for all classes.

### Data set

The selected data sets (Additional File 2: SIGMAR1, HRH1, HERG,and DRD5) were curated using an automated QSAR modelling workflow (https://doi.org/10.1186/s13321-017-0256-5) and divided into two classes using a cut-off activity value (Ki) to separate highly active molecules (Ki<=10.0) as positives and less active and non-active molecules (Ki >10.0) as negatives.

The test example in this tutorial is a small curated dataset retrieved from ChEMBL for the Human Sigma 1 receptor (Q99720). The dataset consists of molecules from several studies measuring the Ki from the displacement of [3H]-pentazocine. About 226 molecules were selected and stored in two separate files, although everything could have been managed with just one. Thus we have a sigma1.smi, containing a SMILES file used for similarity quantification, and a Sigma1\_With\_Class\_labels.csv (Additional File 2), that includes the ChEMBL ids of all molecules, the respective canonical SMILES, the Ki for the assays at hand, and an empirical division in classes.

#### Read curated data

```
dat_full<-read.csv("Sigma1_With_Class_labels.csv")
#################### For data set without class label:
#dat_full$Class[dat_full$Vals<=10]<- "P"
#dat_full$Class[dat_full$Vals>10]<- "N"
#######################################################
dat<-dat_full
head(dat)
```

```
##        chemblId                              CANONICAL_SMILES   Vals Class
## 1 CHEMBL1089752 CN(CCCN1C(=O)[C@H]2Cc3ccccc3CN2C1=O)Cc4ccccc4    7.1     P
## 2 CHEMBL2169891           Clc1ccc(cc1Cl)n2ccc(OCCCN3CCOCC3)n2   16.1     N
## 3 CHEMBL2170045         Cc1cc(OCCN2CCOCC2)n(n1)c3ccc4ccccc4c3 1000.0     N
## 4 CHEMBL2170046 CC(=O)N1CCN(CCOc2cc(C)n(n2)c3ccc4ccccc4c3)CC1   83.4     N
## 5 CHEMBL2170047         Cc1cc(OCCN2CCOCC2=O)nn1c3ccc4ccccc4c3 1000.0     N
## 6 CHEMBL2170048         Cc1cc(OCCCCN2CCOCC2)nn1c3ccc4ccccc4c3   99.1     N
```

```
table(dat$Class)
```

```
## 
##   N   P 
## 170  56
```

### From similarity to distance

Molecular pairwise similarity was quantified using a graph matching algorithm: The Non-contiguous Atom Matching Structural similarity (NAMS). (See NAMS Tutorials: http://www.di.fc.ul.pt/~afalcao/NAMS-tutorials.html). NAMS similarities file for the Sigma 1 (Additional File 2: NAMS\_Sigma1\_sims.txt) Having the SMILES file, we can use the call\_makenamsdb and call\_nams procedures defined in the NAMS Tutorial to make the similarities files. Please take notice that for sigma1.smi Running NAMS mode 2, of all against all involves 25425 comparisons which can take several minutes, depending on the available CPU speeds.

For easier processing and constructing of the similarities matrix, we well need to decoe the ChEMBL IDs into actual matrix row, col numbers, so we will neeed a sort of a Python dictionary, to map ChEMBLID into the internal IDs

```
molids=as.integer(substr(dat$chemblId, 7,200))
NM<-nrow(dat)

#Read NAMS similarity file,
sims<-read.table("NAMS_Sigma1_sims.txt", header=T)

Nsims<-nrow(sims)
D_mols<-1:NM
names(D_mols)<-molids
head(D_mols)
```

```
## 1089752 2169891 2170045 2170046 2170047 2170048 
##       1       2       3       4       5       6
```

```
#calculate the distance matrix,First compute the similarity matrix and afterwards,
smat<-matrix(0,NM,NM)
#check how D_mols is used to convert the chEMBL molids into row and column
for(i in 1:Nsims) smat[D_mols[as.character(sims$molid1[i])], 
                       D_mols[as.character(sims$molid2[i])]]<-sims$Sscore[i]

smat<-smat+t(smat)
#adding the self-similary score of 1.0 in the diagonals concludes the construction of the similarity matrix
diag(smat)<-1.0
smat[1:6, 1:6]
```

```
##        [,1]   [,2]   [,3]   [,4]   [,5]   [,6]
## [1,] 1.0000 0.3519 0.4501 0.4925 0.4678 0.4675
## [2,] 0.3519 1.0000 0.6338 0.5226 0.6303 0.6430
## [3,] 0.4501 0.6338 1.0000 0.7469 0.8997 0.8352
## [4,] 0.4925 0.5226 0.7469 1.0000 0.7808 0.7701
## [5,] 0.4678 0.6303 0.8997 0.7808 1.0000 0.8555
## [6,] 0.4675 0.6430 0.8352 0.7701 0.8555 1.0000
```

```
full_smat<-smat
nrow(full_smat)
```

```
## [1] 226
```

Distance matrix (training data)

```
#split data into train and test
set.seed(200)

smp<-sample(1:NM, NM*.20)
dat<-dat_full[-smp,]
#nrow(dat)
table(dat$Class)
```

```
## 
##   N   P 
## 135  46
```

```
# training data similarity matrix
smat<-full_smat[-smp,-smp]
#nrow(smat)

# Testing/candidate molecules similarity matrix
csmat<-full_smat[smp, -smp]
#nrow(csmat)
#ncol(csmat)

######################################################
#now this is the distance matrix of training data
######################################################
k<-2-(1+sqrt(5))/2;k
```

```
## [1] 0.381966
```

```
dmat<- 1-(k*smat)/(1+k-smat)

#plot similarity vs distance
plot(smat, dmat, ylim=c(0,1), xlab="Similarity", ylab="Distance", main="Similarity to distance tranformations")
```

```
#Final metric space
dmat<- as.dist(dmat)
```

### Projection into a 2D space

```
pcooa<-cmdscale(dmat)

mds<-isoMDS(dmat, trace=F)

samon<-sammon(dmat)

tsne <- Rtsne(as.matrix(dmat), dims = 2, perplexity=30, verbose=TRUE, max_iter = 500, is_distance = TRUE)
```

#### Shepard plots

Since dimensionality reduction is one of the important task in data visualization where it is really necessary to capture the maximum original data information in the new reduced space, Shepard plots were generated to analyze how much molecular initial proximity relationship remained intact. In Shepard plots the original distances are plotted against the projected distances and, ideally, the points(both distances) should lie on a straight line, which would indicate zero distortion in the projection function.

```
#par(mfrow=c(2,2))
sh<- Shepard(dmat, pcooa)
plot(sh, pch = ".", col = "blue", xlab = "Orignal Dissimilarity",  ylab = "Projected Distance(PCooA)", cex.lab= 1.5)
```

```
sh<- Shepard(dmat, mds$points)
plot(sh, pch = ".", col = "blue" ,xlab = "Orignal Dissimilarity",  ylab = "Projected Distance(KMDS)", cex.lab= 1.5)
```

```
sh<- Shepard(dmat, samon$points)
plot(sh, pch = ".", col = "blue",xlab = "Orignal Dissimilarity",  ylab = "Projected Distance(Sammon)", cex.lab= 1.5)
```

```
sh<- Shepard(dmat, tsne$Y)
plot(sh, pch = ".", col = "blue", xlab = "Orignal Dissimilarity",  ylab = "Projected Distance(t-SNE)", cex.lab= 1.5)
```

### Create the probabilistic surface of molecular activity (PSMA)

Generate the surfaces for each class (P, N)

```
# X, Y coordinates frm any DR algorithm can be used.

# X coordinates
X<-pcooa[,1]
#X<-mds$points[,1]
#X<-samon$points[,1]
#X<-tsne$Y[,1]


#Y coordinates
Y<-pcooa[,2]
#Y<-mds$points[,2]
#Y<-samon$points[,2]
#Y<-tsne$Y[,2]

# X, Y coordinates
XYpoints<-pcooa
#XYpoints<-mds$points
#XYpoints<-samon$points
#XYpoints<-tsne$Y

#bandwidth
(bws<-c(bandwidth.nrd(X), bandwidth.nrd(Y)))
```

```
## [1] 0.3596787 0.2621700
```

```
(lims<-c(range(X), range(Y))*2)
```

```
## [1] -0.6300695  0.9055577 -0.5663818  0.5394650
```

```
#kernel density estimation (KDE) is a non-parametric way to estimate the probability 
#density function (describes the relative likelihood for this random variable to take on a 
#given value,it is given by the area under the density function but above the horizontal axis 
img_A<-kde2d(X[dat$Class=="P"], Y[dat$Class=="P"], n=128, lims=lims, h=bws)
img_B<-kde2d(X[dat$Class=="N"], Y[dat$Class=="N"], n=128, lims=lims, h=bws)
```

Now correct the Zs according to the actual priors (the frequencies of each class (P, N))

```
NMM<-length(dat$Class)
priors<-table(dat$Class)/NMM
img_A$z<-img_A$z*priors["P"]
img_B$z<-img_B$z*priors["N"]
```

Here we will show the actual MAP for exceptional quality (class P) and plot all the molecules of each class in separate colors:

- Class P - Red
- Class N - White

```
AP_A<-img_A
AP_A$z<-img_A$z / (img_A$z + img_B$z)
par(mfrow=c(1,1))
image(AP_A, col = terrain.colors(100), main = "Training data projection over 2D probability map (PCooA)")
contour(AP_A, add=T)
points(X[dat$Class=="P"], Y[dat$Class=="P"], pch = 21, bg = "red", col="grey")
points(X[dat$Class=="N"], Y[dat$Class=="N"], pch = 21, bg = "white", col="grey")
```

#### Test/candidate molecules projection

```
#Test/candidate molecules similarity matrix
NCM<- nrow(csmat) 
CD_mols<-molids[smp]
csmat[1:6, 1:6]
```

```
##        [,1]   [,2]   [,3]   [,4]   [,5]   [,6]
## [1,] 0.5363 0.3742 0.5239 0.5868 0.5234 0.5572
## [2,] 0.5548 0.3626 0.4359 0.4279 0.4284 0.4451
## [3,] 0.5602 0.3110 0.4075 0.4128 0.4153 0.4276
## [4,] 0.5232 0.4434 0.4330 0.4000 0.4155 0.4198
## [5,] 0.8059 0.3461 0.4525 0.5106 0.4631 0.4772
## [6,] 0.3873 0.5726 0.4553 0.4361 0.4420 0.4262
```

```
# Test/candidate molecules distance matrix
cdmat<- 1-(k*csmat)/(1+k-csmat)
cdmat[1:6, 1:6]
```

```
##           [,1]      [,2]      [,3]      [,4]      [,5]      [,6]
## [1,] 0.7577668 0.8581698 0.7667872 0.7181247 0.7671454 0.7419493
## [2,] 0.7438063 0.8641304 0.8240091 0.8286877 0.8283976 0.8185300
## [3,] 0.7396128 0.8890801 0.8402703 0.8373080 0.8358994 0.8288616
## [4,] 0.7672886 0.8195505 0.8257142 0.8444076 0.8357864 0.8333455
## [5,] 0.4656404 0.8723788 0.8140442 0.7761769 0.8074927 0.7985400
## [6,] 0.8512712 0.7297715 0.8123281 0.8238911 0.8203882 0.8296718
```

Collocate the test molecules within the molecular space defined by the spatial model.

```
#calculate a data transformation matrix
matC <-  as.matrix(XYpoints)
matD <-  as.matrix(dmat)
matA<- solve(matD) %*% matC

#with the A matrix computed we can now transform the test/candidate distance matrix into the new referenced space

#nrow(cdmat)
#ncol(cdmat)
#nrow(matA)

c_proj<- cdmat %*% matA

Xc<-c_proj[,1]
Yc<-c_proj[,2]
Xc_DR<-Xc
Yc_DR<-Yc
```

Select the molecules that are in higher probability locations for that we may need to define an interpolation function that, for a given image and coordinates is able to retrieve the appropriate surface value With this function it should be quite simple to detect the most promising candidates and attribute them a likelyhood score of being active.

```
get_SurfValue<-function(x, y, img){
  if(x>max(range(img$x))) return(-1)
  if(x<min(range(img$x))) return(-1)
  if(y>max(range(img$y))) return(-1)
  if(y<min(range(img$y))) return(-1)
  (col<-which.min(img$x<x))
  (row<-which.min(img$y<y))
  return(img$z[col, row])
}

NCM<-nrow(csmat)
surf_values<-c()
for(i in 1:NCM) surf_values<-c(surf_values, get_SurfValue(Xc[i], Yc[i], AP_A))


#We can now identify which are the most likely molecules

cands_ords<-order(surf_values, decreasing=T)
mol_id<-CD_mols[cands_ords]
dat_full$id<-as.integer(substr(dat_full$chemblId, 7,200))


pred<-data.frame(dat_full[smp,], prob=surf_values, CD_mols)

#sort predictions
head(pred[order(-pred$prob),], n= 6 )
```

```
##          chemblId                                 CANONICAL_SMILES Vals
## 155 CHEMBL3409378            CN(CCCN1C(=O)Sc2cc(Br)ccc12)Cc3ccccc3  0.6
## 132 CHEMBL3360558             CN(CCCN1c2ccccc2Sc3ccccc13)Cc4ccccc4 28.0
## 139 CHEMBL3360565            CN(CCCN1c2ccccc2Oc3ccccc13)CCc4ccccc4 18.0
## 140 CHEMBL3360566            CN(CCCN1c2ccccc2Sc3ccccc13)CCc4ccccc4 11.0
## 211  CHEMBL365754   CN(CCCN1C(=O)[C@@H]2Cc3ccccc3CN2C1=O)Cc4ccccc4  4.5
## 128 CHEMBL3360554 CN(CCCN1CCc2c([nH]c3ccccc23)C1c4ccccc4)Cc5ccccc5  4.3
##     Class      id      prob CD_mols
## 155     P 3409378 0.4805297 3409378
## 132     N 3360558 0.4754355 3360558
## 139     N 3360565 0.4564916 3360565
## 140     N 3360566 0.4561824 3360566
## 211     P  365754 0.4561824  365754
## 128     P 3360554 0.4526174 3360554
```

```
#write.csv(pred, file = "test_set_predictions.csv", row.names = F)
```

Test set projection over 2D probability map

```
image(AP_A, col = terrain.colors(100), main= "Test set projection over 2D probability map (PCooA)" ) 
contour(AP_A, add=T)

xx_n<-Xc_DR[pred$Class!="P"]
yy_n<-Yc_DR[pred$Class!="P"]

xx_p<-Xc_DR[pred$Class=="P"]
yy_p<-Yc_DR[pred$Class=="P"]

points(xx_n, yy_n, pch = 21, bg = "white", col="grey")
points(xx_p, yy_p, pch = 21, bg = "red", col="grey")
```

#### Probabilistic surface of molecular activity (PSMA)

Convert plot into 3D for visualization.

```
grid_size<-128
color<-terrain.colors(100)
zfacet <- AP_A$z[-1, -1] + AP_A$z[-1, -grid_size] + AP_A$z[-grid_size, -1] + AP_A$z[-grid_size, -grid_size]
facetcol <- cut(zfacet, 100)
pmat<-persp(AP_A, col = color[facetcol], phi = 45, theta = 330, expand=0.3, nticks = 5,ticktype = "simple",  box = T,
            zlab = "Probability", xlab = "X")

xx_n<-Xc_DR[pred$Class!="P"]
yy_n<-Yc_DR[pred$Class!="P"]
zz_n<-surf_values[pred$Class!="P"]

mypoints_n <- trans3d(xx_n, yy_n, zz_n, pmat=pmat)
points(mypoints_n, col="white")
points(mypoints_n, pch=3, col="white")

xx_p<-Xc_DR[pred$Class=="P"]
yy_p<-Yc_DR[pred$Class=="P"]
zz_p<-surf_values[pred$Class=="P"]

mypoints_p <- trans3d(xx_p, yy_p, zz_p, pmat=pmat)
points(mypoints_p, col="red")
points(mypoints_p, pch=3, col="red")
```

### Assessing and validating PSMA as a classification model

PSMA performance assessment using the Area Under Curve (AUC) and the Matthews Coefficient Correlation (MCC).

```
head (pred[order(-pred$prob),])
```

```
##          chemblId                                 CANONICAL_SMILES Vals
## 155 CHEMBL3409378            CN(CCCN1C(=O)Sc2cc(Br)ccc12)Cc3ccccc3  0.6
## 132 CHEMBL3360558             CN(CCCN1c2ccccc2Sc3ccccc13)Cc4ccccc4 28.0
## 139 CHEMBL3360565            CN(CCCN1c2ccccc2Oc3ccccc13)CCc4ccccc4 18.0
## 140 CHEMBL3360566            CN(CCCN1c2ccccc2Sc3ccccc13)CCc4ccccc4 11.0
## 211  CHEMBL365754   CN(CCCN1C(=O)[C@@H]2Cc3ccccc3CN2C1=O)Cc4ccccc4  4.5
## 128 CHEMBL3360554 CN(CCCN1CCc2c([nH]c3ccccc23)C1c4ccccc4)Cc5ccccc5  4.3
##     Class      id      prob CD_mols
## 155     P 3409378 0.4805297 3409378
## 132     N 3360558 0.4754355 3360558
## 139     N 3360565 0.4564916 3360565
## 140     N 3360566 0.4561824 3360566
## 211     P  365754 0.4561824  365754
## 128     P 3360554 0.4526174 3360554
```

```
table(pred$Class)
```

```
## 
##  N  P 
## 35 10
```

```
truth<-pred$Class


###################################
# Area Under Curve (AUC)
plot.roc(truth, pred$prob,print.auc=T, 
         print.thres=T,
         print.thres.pch=25,
         print.thres.adj=c(0.5,2),
         print.thres.col="blue",
         print.thres.cex=0.8,
         print.auc.adj=c(0,1),
         print.auc.col="red",
         print.auc.cex=1) 
title("ROC Curve", line = 2.5)
```

```
preds<-c("N", "P")[(pred$prob>0.349)+1];
tt1<-table(truth, preds);tt1
```

```
##      preds
## truth  N  P
##     N 26  9
##     P  0 10
```

```
TP<-tt1[2,2]
TN<-tt1[1,1]
FP<-tt1[1,2]
FN<-tt1[2,1]

# Matthews Coefficient Correlation (MCC)
MCC<-mcc(TP=TP, FP=FP, TN=TN, FN=FN);MCC
```

```
## [1] 0.6252819
```
